# Assessment of dynamic functional connectivity in resting‐state fMRI using the sliding window technique

**DOI:** 10.1002/brb3.1255

**Published:** 2019-03-18

**Authors:** Antonis D. Savva, Georgios D. Mitsis, George K. Matsopoulos

**Affiliations:** ^1^ School of Electrical and Computer Engineering National Technical University of Athens Athens Greece; ^2^ Department of Bioengineering McGill University Montreal Quebec Canada

**Keywords:** functional connectivity metrics, information based metrics, Multiplication of Temporal Derivatives, partial correlation metrics, surrogate data, window size

## Abstract

**Introduction:**

Recent studies related to assessing functional connectivity (FC) in resting‐state functional magnetic resonance imaging have revealed that the resulting connectivity patterns exhibit considerable fluctuations (dynamic FC [dFC]). A widely applied method for quantifying dFC is the sliding window technique. According to this method, the data are divided into segments with the same length (window size) and a correlation metric is employed to assess the connectivity within these segments, whereby the window size is often empirically chosen.

**Methods:**

In this study, we rigorously investigate the assessment of dFC using the sliding window approach. Specifically, we perform a detailed comparison between different correlation metrics, including Pearson, Spearman and Kendall correlation, Pearson and Spearman partial correlation, Mutual Information (MI), Variation of Information (VI), Kullback–Leibler divergence, Multiplication of Temporal Derivatives and Inverse Covariance.

**Results:**

Using test–retest datasets, we show that MI and VI yielded the most consistent results by achieving high reliability with respect to dFC estimates for different window sizes. Subsequent hypothesis testing, based on multivariate phase randomization surrogate data generation, allowed the identification of dynamic connections between the posterior cingulate cortex and regions in the frontal lobe and inferior parietal lobes, which were overall in agreement with previous studies.

**Conclusions:**

In the case of MI and VI, a window size of at least 120 s was found to be necessary for detecting dFC for some of the previously identified dynamically connected regions.

## 1 INTRODUCTION

Functional magnetic resonance imaging (fMRI) is perhaps the primary imaging technique employed for investigating the function of the human brain. One of the main reasons is its excellent spatial resolution and noninvasive nature, as compared to other imaging methodologies, such as positron emission tomography (PET) and single‐photon emission computed tomography (SPECT). In addition, fMRI provides a good balance between spatial resolution for localization of activations in the brain as well as continuously increasing temporal resolution, as compared to magnetoencephalography (MEG) and electroencephalography (EEG) (Huettel, Song, & McCarthy, 2008). Over the past years, considerable attention has been shifted to studying the functional organization of the brain in the absence of explicit tasks. Through this approach, which is commonly referred to as resting‐state fMRI (rs‐fMRI), it has become possible to draw conclusions about brain function under healthy and pathological conditions, involving but not limited to, the discovery of networks comprised by distant brain regions and changes induced by neurological and other disorders (Buckner & Vincent, 2007; Damoiseaux et al., 2006; De Luca, Beckmann, De Stefano, Matthews, & Smith, 2006; Rombouts, Barkhof, Goekoop, Stam, & Scheltens, 2005; Shehzad et al., 2009; Xia et al., 2013). During the resting‐state condition, it has been consistently found that particular brain regions activate consisting the well‐known Default Mode Network (DMN) (Damoiseaux et al., 2006; Raichle et al., 2001). As a result, the DMN is often the subject of investigation in both healthy (Andrews‐Hanna, Reidler, Sepulcre, Poulin, & Buckner, 2010; Christoff, Gordon, Smallwood, Smith, & Schooler, 2009; Fransson & Marrelec, 2008; Greicius, Krasnow, Reiss, & Menon, 2003) and pathological conditions (Buckner, Andrews‐Hanna, & Schacter, 2008; Horne & Norbury, 2018; Jiang et al., 2018; Padmanabhan, Lynch, Schaer, & Menon, 2017; Whitfield‐Gabrieli & Ford, 2012), expanding our knowledge regarding functional organization of the resting human brain and how is differentiated in nonhealthy conditions (Smith, Vidaurre et al., 2013; Zhang, Shen, & Lin, 2018).

Within the framework of rs‐fMRI, it is often customary to apply functional connectivity (FC) analysis for quantifying the statistical associations or dependencies of spatially distinct and temporally correlated brain regions (Friston, 2011; Sakoğlu et al., 2010). Functional connectivity was initially assessed under the assumption of stationarity, which assumes that the underlying connections do not change over time (Hutchison, Womelsdorf, Allen et al., 2013; Preti, Bolton, & Van De Ville, 2017). However, recent advances in neuroimaging have highlighted the fact that FC between brain regions is in fact dynamic, suggesting that the statistical properties of the corresponding correlation measures are subject to change over different time scales (Calhoun & Adali, 2016; Chang & Glover, 2010; Hutchison, Womelsdorf, Allen et al., 2013; Preti et al., 2017). This newly adopted approach yields promise for better understanding the nature of resting‐state activity and may provide new insights concerning a variety of brain conditions (Damaraju et al., 2014; Leonardi, Richiardi, & Van De Ville, 2013; Li et al., 2014).

Over the past years, several approaches have been employed to quantify resting‐state dynamic functional connectivity (rs‐dFC). These can be divided into two main categories: time domain analysis and time‐frequency joint mapping. The former includes the detection of coactivation patterns (Liu & Duyn, 2013), the discovery of repeatable spatiotemporal patterns (Majeed et al., 2011), as well as the “temporal functional mode” approach which is based on temporal independent components analysis (Smith et al., 2012). However, the most common approach to assess rs‐dFC is by far the sliding window approach, whereby the fMRI data are segmented in (possibly overlapping) windows and functional interconnections between different brain areas are assessed within each window (Allen et al., 2014; Barttfeld et al., 2015; Choe et al., 2017; Handwerker, Roopchansingh, Gonzalez‐Castillo, & Bandettini, 2012; Hutchison, Womelsdorf, Gati, Everling, & Menon, 2013).

On the other hand, assessing rs‐dFC in the time‐frequency domain provides a way to quantify correlations between Blood Oxygen Level Dependent (BOLD) signals in different brain areas as a function of both time and frequency. So far, this approach has been implemented using Wavelet Transform Coherence (WTC), which decomposes the BOLD time‐series into multiple scales (Chang & Glover, 2010; Torrence & Compo, 1998). Therefore, it provides a framework for capturing correlations between slower/faster fluctuations present in rs‐fMRI data. Despite the advantages of the time‐frequency approach, relatively few studies have utilized it according to a recent literature review (Preti et al., 2017). On the other hand, the vast majority of rs‐dFC studies have employed the sliding window technique, mainly due to its simplicity (Preti et al., 2017).

The sliding window technique requires that certain parameters should be selected a priori: (a) the length of the window; (b) the FC metric; (c) the step of window shifting; and (d) the weighting scheme for the data within each segment. The first choice concerns the selection of the duration of the window. This is a crucial parameter, as it determines the tradeoff between time resolution and estimation accuracy; specifically, a small window size yields improved capability to track fast the temporal changes but at the cost of introducing spurious fluctuations and increased sensitivity to noise (Leonardi & Van De Ville, 2015; Shakil, Billings, Keilholz, & Lee, 2018; Shakil, Keilholz, & Chin‐Hui, 2015). Most related studies have empirically converged to window size values between 30 and 60 s, while some have considered larger values—up to 240 s (Hutchison, Womelsdorf, Allen et al., 2013; Hutchison, Womelsdorf, Gati et al., 2013; Preti et al., 2017). Furthermore, a critical step for applying sliding window analysis is the choice of FC metric for calculating the statistical interdependencies between the time‐series within each window. So far, the most frequently used metrics for quantifying rs‐dFC are the Pearson linear correlation and the covariance matrix, while other metrics have been used less frequently, such as Spearman rank correlation and Multiplication of Temporal Derivatives (MTD) (Damaraju et al., 2014; Hindriks et al., 2016; Preti et al., 2017; Shine et al., 2015). The step of window shifting, that is, the number of time lags by which the sliding window is shifted is commonly selected as one time lag (1 TR) (Allen et al., 2014; Hutchison, Womelsdorf, Allen et al., 2013; Hutchison, Womelsdorf, Gati et al., 2013). Different windowing types, which weigh the data inside each segment can be also applied, aiming to minimize the impact of noisy observations, which could result in abrupt alterations in the corresponding rs‐dFC time‐series (Lindquist, Xu, Nebel, & Caffo, 2014; Preti et al., 2017; Zalesky, Fornito, Cocchi, Gollo, & Breakspear, 2014). Commonly used window functions include the Hamming, Hanning and Gaussian functions, while the choice of window functions is reviewed in detail in Preti et al. (2017). Nevertheless, a number of studies employ the simple rectangular window, with an increasing number of new studies using weighted variants, for example, Gaussian and tapered windows (Preti et al., 2017).

The application of the sliding window methodology results in time‐series that contain the selected correlation metric values within each window. However, these values are the estimates of the true FC and thus are subject to statistical ambiguity (Hindriks et al., 2016). Therefore, a proper statistical framework should be applied to determine whether the observed variation in the FC metric values can be characterized as dynamic functional connectivity (dFC) (Hindriks et al., 2016). To this end, a commonly used approach is to generate surrogate data from the initial measurements to formulate the null hypothesis (stationary FC) and conclude whether any given FC time‐series exhibits dFC, that is, this null hypothesis can be rejected (Chang & Glover, 2010; Hindriks et al., 2016; Zalesky et al., 2014).

Despite the widespread application of the sliding window approach, it has been suggested that a gold standard is currently absent in the context of assessing dFC in rs‐fMRI (Sadia Shakil, Lee, & Keilholz, 2016). In this latter study, simulated resting‐state networks were constructed in order to evaluate sliding window parameters such as window length, offset, type and choices regarding noise and filtering, while employing Pearson linear correlation as the FC metric. Their simulation study suggested that the detection of transitions between different brain states is highly dependent on the window length and offset (Sadia Shakil et al., 2016).

In this context, the main aim of the current study was to rigorously investigate the application of the sliding window technique to detect dFC in experimental rs‐fMRI data, focusing on the choice of the FC metric and the size of the window used. In particular, we consider a wide range of correlation metrics, some of which, to our knowledge, have not been employed in previous rs‐fMRI studies. Moreover, we thoroughly examine the effect of window size on each examined metric, aiming to identify the sensitivity of each metric to the choice of window size. We use publicly available data from the Human Connectome Project (Smith, Beckmann et al., 2013), collected from 100 healthy controls and divided into two groups of 50 subjects each to form a test–retest validation scheme. A statistical testing framework, based on generation of surrogate data using the multivariate phase randomization (MVPR) and multivariate auto‐regressive (MVAR) approaches, was employed, for assessing the presence of dFC between regions of the Default Mode Network, for all FC metrics and window sizes.

## 2 MATERIALS AND METHODS

**Figure 1:**
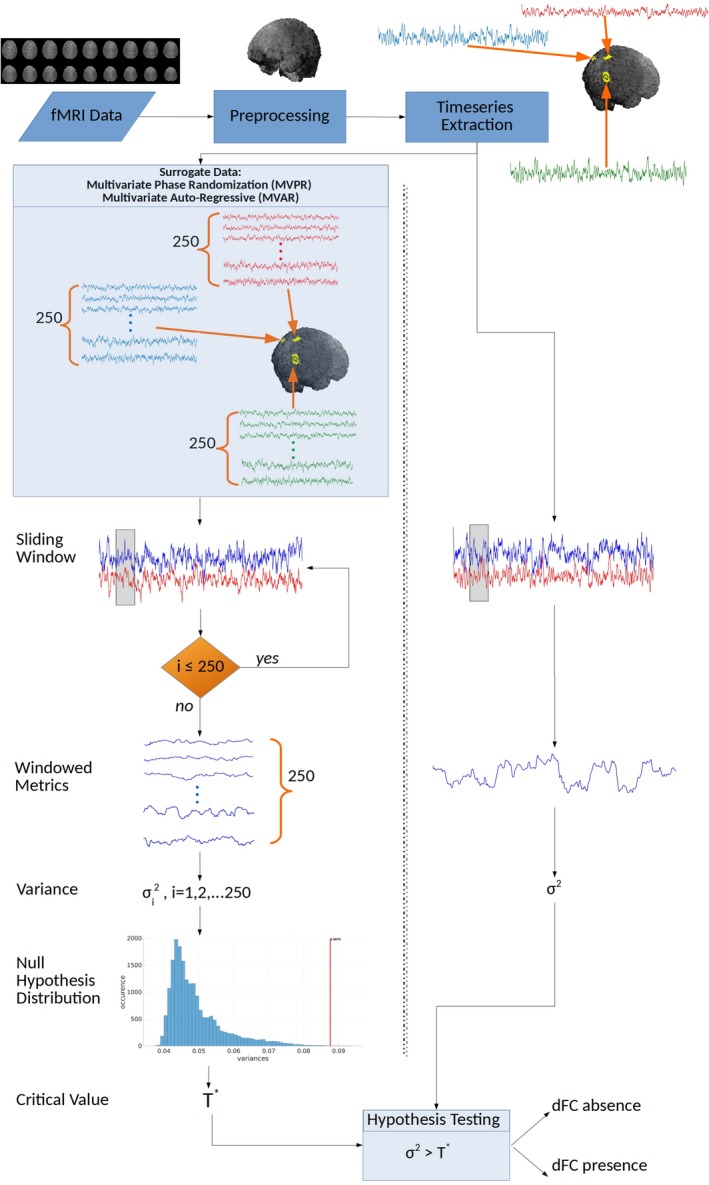
Overview of the examined procedure for assessing dynamic functional connectivity (dFC) using the sliding window method

The flow chart in Figure 1 illustrates the procedure that we followed. Below, we provide a detailed description regarding the employed data and their preprocessing, as well as the approach for extracting time‐series from specific brain regions. Subsequently, a comprehensive description is provided for producing surrogate data and the various variations of the sliding window technique that we examined. Finally, the construction of suitable null hypothesis histograms is described, along with the resulting hypothesis testing schemes for dFC assessment.

### 2.1 Data acquisition and preprocessing

Resting‐state fMRI data from 100 subjects (41 males, 59 females) were retrieved from the Human Connectome Project (HCP) initiative (S900 release) (Smith, Beckmann et al., 2013). Individuals were instructed to keep their eyes open with relaxed fixation on a projected bright cross‐hair on a dark background. Data were acquired using a customized Siemens scanner at 3T using a gradient‐echo EPI sequence, TR/TE 720/33.1ms, flip angle 52^°^, field of view $208\times 180\;{\mathrm{mm}}^{2}$, matrix $104\times 90\;{\mathrm{mm}}^{2}$, voxel dimensions $2\times 2\times 2\;{\mathrm{mm}}^{3}$, multiband factor of 8, echo spacing 0.58ms, and BW 2290Hz/Px covering a period of 14 min and 33 s yielding a total of 1,200 volumes. In the present study, only the right–left encoded data from the first session were analyzed. To perform a test–retest analysis, the dataset was divided into two groups each one consisting of 50 subjects. Hereafter, the terms “Dataset A” and “Dataset B” are used interchangeably with “test” and “retest,” respectively.

The minimally preprocessing pipeline was adopted (Glasser et al., 2013). This procedure consists of eliminating spatial distortions due to gradient nonlinearities, correction of head motion by aligning functional data to the single band reference image using 6 *df*, correction for distortion induced by the B0 field, boundary based registration to the T1 weighted structural image and alignment to the 2mm montreal neurological institute (MNI) space using nonlinear registration. All the above mentioned transformations are concatenated and applied to the raw data using a single spline interpolation scheme in order to reduce blurring effects. The final steps include global (four‐dimensional) intensity normalization to a value of 10,000, as well as smoothing using a 2mm FWHM geodesic Gaussian procedure. An additional step of high‐pass temporal filtering at 0.0067Hz (corresponding to a cutoff time of 150 s) was also performed in order to remove any slow drifts and trends present in the data (Smith, Beckmann et al., 2013). As a result, in all time‐series, frequencies f>0.0067Hz were present and were common for all considered metrics, window sizes and surrogate data methods. In the study of Smith, Beckmann et al., 2013, it was suggested that high‐pass temporal filtering at f=0.0005Hz(2000s) is adequate for removing linear trends (Smith, Beckmann et al., 2013). We also considered this option; however, in some cases higher order trends (e.g., 2nd, 3rd order) were still present in the data, which were removed using cutoff frequency of f=0.0067Hz(150s). A representative example is illustrated in Figure S1 (Supporting information).

### 2.2 Time‐series extraction

We focused on the brain regions comprising the Default Mode Network (DMN). To this end, we used the Dorsal and Ventral DMN functional masks (http://findlab.stanford.edu/functional_ROIs.html) to extract BOLD time‐series (Shirer, Ryali, Rykhlevskaia, Menon, & Greicius, 2012). Both masks were merged into a new functional mask comprising of 13 regions of interest (ROIs). All 13 regions are listed in Table 1 along with their abbreviations and coordinates in MNI space. Then, the mean BOLD time‐series across all voxels inside each ROI was calculated, thus yielding 13 time‐series for each subject.

**Table 1:** Nomenclature, abbreviations, and cluster centroids of the considered brain ROIs

Region name	Abbr.	Coordinates in MNI (mm)		
		*x*	*y*	*z*
Cerebellum	Cer	14	–46	–52
Left hippocampus	L‐Hipp	–24	–30	–14
Right hippocampus	R‐Hipp	26	–24	–16
Medial prefrontal cortex	mPFC	0	52	14
Thalamus	Thal	0	–10	6
Posterior cingulate cortex	PCC	0	–54	30
Right inferior parietal	R‐IP	44	–72	32
Left inferior parietal	L‐IP	–36	–80	32
Left inferior parietal–2	L‐IP(2)	–48	–66	34
Anterior cingulate gyrus	ACG	0	–14	36
Right middle frontal gyrus	R‐MFG	28	32	44
Precuneus	Prec	0	–56	56
Left middle frontal gyrus	L‐MFG	–26	14	52

Note

ROI: region of interest.

### 2.3 Surrogate data

Since the ground truth regarding the presence of dynamic connections between different brain regions is not known, one cannot derive conclusions by simply observing the values obtained by the application of sliding window technique, as spurious fluctuations may be introduced due to the use of finite data samples (Hindriks et al., 2016; Hutchison, Womelsdorf, Gati et al., 2013; Leonardi & Van De Ville, 2015). Therefore, a statistical framework is needed for assessing whether a particular pair of brain regions exhibits time‐dependent fluctuations (dFC). The most common approach for addressing this issue is the construction of surrogate data from the initial BOLD recordings (Pereda, Quiroga, & Bhattacharya, 2005; Schreiber & Schmitz, 2000). Surrogate data typically aim to preserve basic properties that the original data exhibit, for example, auto‐covariance sequence, stationary cross‐correlation, power spectral density, cross power spectral density, and amplitude distribution (Pereda et al., 2005; Prichard & Theiler, 1994; Schreiber & Schmitz, 2000; Zalesky et al., 2014). In the context of FC studies, surrogate data are also generated under the assumption of stationary FC (Hindriks et al., 2016; Liégeois, Laumann, Snyder, Zhou, & Yeo, 2017). In the present study, to examine whether a ROI pair exhibits time‐varying FC, two surrogate data methods were employed: MVPR and MVAR models (Hindriks et al., 2016; Zalesky et al., 2014). Below, a description is provided for both methods.

Phase randomization consists of the following procedure for producing surrogate data (Prichard & Theiler, 1994): Let $x=[x_{1},\;x_{2},\;\cdots ,\;x_{n}]$ denote the BOLD recordings from n=13 brain regions, each one of them comprising of $\tilde{T}=1200$ time points and $X=[X_{1},\;X_{2},\;\cdots ,\;X_{n}]$ denote their discrete Fourier transform. Next, a uniformly distributed random phase $\left(\varphi =\left[\varphi_{1},\varphi_{2},\cdots ,\varphi_{\tilde{T}}\right]\right)$, in the interval [0,2π] is generated and applied to each transformed signal as follows: ${\hat{X}}_{k}=X_{k}e^{i\varphi },\;k=1,2,\;\cdots ,\;n$. This suggests that all signals in the frequency domain are multiplied by the same uniformly random phase (Hindriks et al., 2016). Finally, the inverse Fourier transform is calculated and one surrogate copy is obtained. This procedure was applied for obtaining a total of 250 randomized copies for each subject (Hindriks et al., 2016; Zalesky et al., 2014).

In the case of the autoregressive‐based approach, the multivariate version was favored over its bivariate alternative, as the latter may introduce a large number of significant connections (false positives) (Liégeois et al., 2017). Autoregressive models represent the output of a random variable as a linear combination of its own previous values (Efron & Tibshirani, 1986). Multivariate Auto‐Regressive models represent a set of signals as a combination of both their own past values as well as the past values of all other signals in the set (Lütkepohl, 2005). The weighting of the effect of past signal values is given by Equation (1), where a stationary MVAR model was initially fitted to the BOLD time‐series:

(1) $$\begin{array}{c} x_{1}\left(t\right)=\sum_{i=1}^{p}a_{i}^{x_{1}}x_{1}\left(t-i\right)+\sum_{i=1}^{p}b_{i}^{x_{1}}x_{2}\left(t-i\right)+\ldots +\sum_{i=1}^{p}w_{i}^{x_{1}}x_{n}\left(t-i\right)+\varepsilon^{x_{1}}\left(t\right)\\ x_{2}\left(t\right)=\sum_{i=1}^{p}a_{i}^{x_{2}}x_{1}\left(t-i\right)+\sum_{i=1}^{p}b_{i}^{x_{2}}x_{2}\left(t-i\right)+\ldots +\sum_{i=1}^{p}w_{i}^{x_{2}}x_{n}\left(t-i\right)+\varepsilon^{x_{2}}\left(t\right)\\ \;\;\;\vdots \\ x_{n}\left(t\right)=\sum_{i=1}^{p}a_{i}^{x_{n}}x_{1}\left(t-i\right)+\sum_{i=1}^{p}b_{i}^{x_{n}}x_{2}\left(t-i\right)+\ldots +\sum_{i=1}^{p}w_{i}^{x_{n}}x_{n}\left(t-i\right)+\varepsilon^{x_{n}}\left(t\right) \end{array}$$

where n=13.

Moreover, Equation (1) can be written in matrix notation as follows:

(2) $$x\left(t\right)=A_{1}x\left(t-1\right)+A_{2}x\left(t-2\right)+\cdots +A_{p}x\left(t-p\right)+\varepsilon \left(t\right)$$

where: $x={\left[x_{1},\;x_{2},\;\cdots ,\;x_{n}\right]}^{T}$ are the simultaneously recorded BOLD time‐series, $\varepsilon ={\left[\varepsilon^{x_{1}},\;\varepsilon^{x_{2}},\;\cdots ,\;\varepsilon^{x_{n}}\right]}^{T}$ expresses the residuals after model fitting and $A_{i}=\left[\begin{array}{cccc} a_{i}^{x_{1}} & b_{i}^{x_{1}} & \cdots & w_{i}^{x_{1}}\\ a_{i}^{x_{2}} & b_{i}^{x_{2}} & \cdots & w_{i}^{x_{2}}\\ \vdots & \vdots & \ddots & \vdots \\ a_{i}^{x_{n}} & b_{i}^{x_{n}} & \cdots & w_{i}^{x_{n}} \end{array}\right]i=1,2,\;\cdots ,\;p$, is the coefficients matrix.

The polynomial order p defines the number of past signal values that is considered in the MVAR model. We selected the value of p based on the minimization of the Schwarz Bayesian Criterion (SBC) (Zalesky et al., 2014). Having estimated the coefficients matrix $A_{i}$, we generated randomized copies closely following the procedure illustrated in previous studies (Chang & Glover, 2010; Zalesky et al., 2014). In particular, the following steps were implemented:

- Step 1

Choose a random time point $t_{0}$ from the uniform distribution, satisfying $1\leq t_{0}\leq \tilde{T}-p.$

- Step 2

Initialize a surrogate copy $x_{s}\left(t\right)=x\left(\hat{t}\right)$, where t=1,2,⋯,p and $\hat{t}=t_{0},t_{0}+1,\cdots ,t_{0}+p-1$. This step essentially sets the first p values of the surrogate copy to be a set of p adjacent values from the initial time‐series.

- Step 3

For t=p+1,⋯,T choose a random time point $\tilde{t}$ uniformly $\left(1\leq \tilde{t}\leq T-p\right)$ and let $\tilde{\varepsilon }\left(t\right)=\varepsilon \left(\tilde{t}\right)$. Through this step a new set of residuals is formed by randomly sampling the residuals of the model (Equation 2).

- Step 4

Set $x_{s}\left(t\right)=A_{1}x_{s}\left(t-1\right)+A_{2}x_{s}\left(t-2\right)+\cdots +A_{p}x_{s}\left(t-p\right)+\tilde{\varepsilon }$(*t*).

Following steps 1–4, a single randomized copy is generated. Again, a total number of 250 randomized copies were created for each subject (Hindriks et al., 2016; Zalesky et al., 2014). Concerning the properties of initial data, the MVPR approach better preserved auto‐covariance sequence, stationary cross‐correlation, power spectral density, cross power spectral density, and amplitude distribution compared to MVAR method, as illustrated in Figure S2 in the Supporting information; therefore we focus on the MVPR‐obtained results.

### 2.4 Sliding window methodology

The sliding window methodology considers each pair of time‐series corresponding to the above mentioned ROIs and calculates a FC metric in each segment of the examined BOLD signals, resulting in a collection of windowed metric values. After the estimation of the windowed metrics for all region pairs, the resulting values were assembled in a three‐dimensional matrix (regions×regions×windows) whereby the variance of the windowed metrics, which was subsequently used to assess dFC, was calculated across the third dimension. In the current study, a rectangular window was employed, shifted by one time point (1 TR).

### 2.5 Employed metrics

One of the main aims of this study was to examine the performance of wide range of linear (Pearson full and partial correlation and Inverse Covariance [ICOV]) and nonlinear (Spearman full and partial correlation, Kendall correlation, Mutual Information (MI), Variation of Information (VI), Kullback–Leibler divergence, MTD) metrics for assessing rs‐dFC, compared to previous studies. A brief description of the employed metrics is presented below.

#### 2.5.1 Pearson linear correlation

Pearson correlation coefficient (ρ) is a linear, commonly used metric in FC studies (Preti et al., 2017). It expresses the linear dependence or association between two random variables X and Y as:

(3) $$\rho \left(X,Y\right)=\frac{E\left[\left(X-\bar{\mu_{X}}\right)\left(Y-\bar{\mu_{Y}}\right)\right]}{\sigma_{X}\sigma_{Y}}$$

where E indicates the expected value while ($\bar{\mu_{X}}$, $\sigma_{X})$ and $\left(\bar{\mu_{Y}},\sigma_{Y}\right)$ denote the mean and standard deviation values of random variables X and Y, respectively.

#### 2.5.2 Pearson partial correlation

Partial correlations can be advantageous in cases where the desired measure is the degree of correlation between two random variables after removing the effect of all other variables, usually through linear regression (Smith et al., 2011). Let X,Y be the two random variables and Z be the set of variables whose effect must be removed from X andY. Initially, a linear regression step is performed between X and Z **,** as well as between Y and Z as:

(4) $$\begin{array}{c} X=\beta_{X}Z+\varepsilon_{X}\\ Y=\beta_{Y}Z+\varepsilon_{Y} \end{array}$$

After calculating the regression coefficients ${(\beta }_{X},\beta_{Y})$ and residuals ${(\varepsilon }_{X},\varepsilon_{Y})$, the Pearson partial correlation is obtained by calculating the Pearson linear correlation of the residuals, that is, by assessing $\rho (\varepsilon_{X},\varepsilon_{Y})$ according to Equation (3).

#### 2.5.3 ICOV representation

An alternative linear metric, commonly employed in FC analyses, is the ICOV matrix which is also referred to as the precision matrix. However, earlier studies have suggested that direct computation of the covariance matrix by matrix inversion is an ill‐posed problem, especially in cases where the number of data points in the considered time‐series is comparable to the number of brain region connections. This yields a poor estimate which may diverge from the real covariance matrix (Varoquaux & Craddock, 2013). To tackle this problem, an iterative optimization procedure based on Ledoit–Wolf shrinkage assessment has been suggested for directly estimating the precision matrix and has been reported to achieve superior performance compared to standard matrix inversion (Ledoit & Wolf, 2004; Varoquaux & Craddock, 2013). The optimization procedure consists of applying a cost function in the form of a $L_{1}$ norm to the precision matrix, in order to enforce a small number of coefficients to be nonzero. This cost is controlled by a regularization parameter λ which was set to 0.1 in the present study, following (Barttfeld et al., 2015).

#### 2.5.4 Spearman rank correlation

The Spearman correlation coefficient $\left(\rho_{s}\right)$ is a nonlinear metric quantifying the rank interrelationship between two random variables X and Y (Thompson & Fransson, 2015). Its estimation consists of calculating the Pearson linear correlation between the ranked variables rX and rY, as obtained by arranging the values of each random variable in ascending order and assigning rank labels first, second, third to each of them. Subsequently, $\rho_{s}$ can be calculated as:

(5) $$\rho_{s}\left(X,Y\right)=\frac{E\left[\left(rX-\bar{\mu_{rX}}\right)\left(Y-\bar{\mu_{rY}}\right)\right]}{\sigma_{rX}\sigma_{rY}}$$

where ($\bar{\mu_{rX}}$, $\sigma_{rX})$ and $\left(\bar{\mu_{rY}},\sigma_{rY}\right)$ indicate the mean and standard deviation values of the ranked variables rX and rY, respectively.

#### 2.5.5 Spearman partial correlation

Similar to Pearson partial correlation, the Spearman partial correlation quantifies the rank association between two random variables, when the effects of all the remaining ones have been regressed out (Smith et al., 2011). Similar to the procedure described above for Pearson partial correlation, a linear regression step was performed as shown in Equation (4) and, subsequently, the residuals were utilized for calculating the Spearman partial correlation $\rho_{s}\left(\varepsilon_{X},\varepsilon_{Y}\right)$ (Equation 5).

#### 2.5.6 Kendall correlation

The Kendall correlation $\left(\tau \right)$ is another nonlinear metric for assessing rank equivalence (Kendall, 1938). In order to perform the calculation, the values of the random variables X and Y are arranged in pairs: $\left(x_{i},y_{i}\right),i=1,2,\cdots ,N$, where N is the total number of observations. Subsequently, a comparison is conducted between all pairs $\left(x_{i},y_{i}\right)$ and $\left(x_{j},y_{j}\right)$ with i≠j, in order to conclude whether pairs can be labeled as *concordant*,* discordant* or *none of those*. The conditions for such characterization are:

- If $\left\{{(x}_{i}>x_{j})\;AND\;{(y}_{i}>y_{j})\right\}OR\left\{{(x}_{i}<x_{j})\;AND\;{(y}_{i}<y_{j})\right\}$, the pairs are *concordant*. The total number of concordant pairs is denoted as $N_{C}$.
- If $\left\{{(x}_{i}>x_{j})\;AND\;{(y}_{i}<y_{j})\right\}OR\left\{{(x}_{i}<x_{j})\;AND\;{(y}_{i}>y_{j})\right\}$, the pairs are *discordant*. The total number of concordant pairs is denoted as $N_{D}$.
- If ${(x}_{i}=x_{j})\;AND\;{(y}_{i}=y_{j})$, the pairs cannot be classified as concordant or discordant and therefore are not considered in Kendall's τ calculation.

Finally, Kendall correlation is computed as:

(6) $$\tau \left(X,Y\right)=\frac{2(N_{C}-N_{D})}{N(N-1)}$$

#### 2.5.7 Mutual information

The MI between two random variables $\left(X,Y\right)$ can be defined as the amount of information shared by X and Y, as shown in Equation (7). Mutual Information is a nonlinear metric and is commonly measured in bits (Brown, Pocock, Zhao, & Luján, 2012).

(7) $$I\left(X,Y\right)=\sum_{x\in X}\sum_{y\in Y}p\left(x,y\right)\log_{2}\left(\frac{p(x,y)}{p\left(x\right)p\left(y\right)}\right)$$

where p(x,y) and $p\left(x\right),\;p\left(y\right)$ denote the joint and marginal probability density functions (PDFs) of Xand Y, respectively. The corresponding PDFs were estimated using histogram estimators with number of bins equal to the total number of time points (Brown et al., 2012). Following histogram assessment, the probability of each observed value was calculated as the corresponding frequency of occurrence. Finally, a normalization by the total number of time points was performed (Brown et al., 2012).

#### 2.5.8 Variation of information

The VI is a newly introduced nonlinear metric, initially utilized for estimating the distance between two partitions (clusterings) of the same dataset (Meilă, 2007). In our case, this metric was employed for estimating the distance between two random variables, X,Y, as shown below (Meilă, 2007):

(8) $$VI\left(X,Y\right)=H\left(X\right)+H\left(Y\right)-2I\left(X,Y\right)$$

where $H\left(X\right)$ is the entropy of X calculated as: $H\left(X\right)=\sum_{i=1}^{N}p\left(x_{i}\right)\log_{2}p\left(x_{i}\right)$. The entropy of Y is calculated in a similar manner, while $p\left(x_{i}\right)$ expresses the probability of observing the value $x_{i}$ and was calculated as mentioned above for MI metric.

#### 2.5.9 Kullback–Leibler divergence

The Kullback–Leibler (KL) divergence between two random variables X and Y is a measure of the similarity between their respective PDFs (Kullback, 1997). By denoting p(x) and p(y) the PDFs of X and Y **,** respectively, the KL divergence can be estimated as:

(9) $$\mathrm{KL}\left(X\|Y\right)=\sum_{i=1}^{N}p\left(x\right)\log_{2}\frac{p(x)}{p(y)}$$

As can be seen from the above definition, the Kullback–Leibler divergence is not symmetric since KL(X‖Y)≠KL(Y‖X). In order to overcome this issue, a symmetrized version, which was utilized in the present study, has been proposed (Johnson & Sinanovic, 2001):

(10) $${\text{KL}}_{s}\left(X\|Y\right)=\frac{\text{KL}(X\|Y)+\text{KL}(Y\|X)}{2}$$

#### 2.5.10 Multiplication of temporal derivatives

Multiplication of temporal derivatives is a recently proposed nonlinear metric for quantifying dFC and involves calculating the temporal derivative (dt) and standard deviation of the derivative $\left(\sigma_{\mathrm{dt}}\right)$ of the examined time‐series (Shine et al., 2015). These quantities are then combined in order to obtain the final metric by averaging in a windowed manner over time as:

(11) $${\mathrm{MTD}}_{\mathrm{ijt}}=\frac{1}{2w+1}\sum_{k=t-w}^{t+w}\frac{dt_{\mathrm{ik}}\times dt_{\mathrm{jk}}}{\sigma_{i}\times \sigma_{j}}$$

where i,j represent the brain regions, t expresses the time, and w is the window length.

### 2.6 Window length

The window length is a crucial parameter, which may considerably affect the final results (Hutchison, Womelsdorf, Allen et al., 2013; Hutchison, Womelsdorf, Gati et al., 2013). There is still a debate concerning the optimal value of window length (Preti et al., 2017). Therefore, we have rigorously examined the effect of window size by considering window sizes between 20 and 150 s with a step of 10 s, for balancing between sufficient number of window sizes, that is, 14, and the increased processing time for deriving null hypothesis distributions.

### 2.7 Null hypothesis distribution

To proceed to hypothesis testing, histograms of the variance of windowed metric values corresponding to the null hypothesis (stationary FC) were constructed using the surrogate data. This initially yielded a collection of variances of windowed metrics, corresponding to a regions×regions×subjects×surrogates (13 × 13×100 × 250) matrix. Subsequently, the average across subjects was calculated yielding a 13 × 13 × 250 matrix, defining a null distribution for each region pair (Hindriks et al., 2016). Specifically, 78 distributions were generated, since $\left(\begin{array}{c} 13\\ 2 \end{array}\right)=78$. A previous study proposed aggregating all individual null distributions into a single highly resolved distribution and subsequently performing hypothesis testing (Zalesky et al., 2014). In the present study, both options were examined, that is, a null distribution for each pair and a null distribution for all pairs. This procedure was repeated for all employed FC metrics and window lengths.

### 2.8 Hypothesis testing

As illustrated in the previous section, through surrogate data analysis, it is possible to define the distribution of the null hypothesis and then perform hypothesis testing for assessing the presence of dFC. The resulting statistical hypothesis can be formally expressed as:

(12) $$\begin{array}{c} H_{0}:\;\text{dFC}\;\text{absence}\\ H_{1}:\;\text{dFC}\;\text{presence} \end{array}$$

In the current study, the variance $\left(\sigma^{2}\right)$ of windowed metrics is considered as a measure of dFC; therefore, the hypothesis testing can be expressed as follows (Choe et al., 2017):

(13) $$\begin{array}{c} H_{0}:\;\sigma^{2}=0\\ H_{1}:\;\sigma^{2}>0 \end{array}$$

Independent of the chosen null distribution mode (null distribution for each pair vs. all pairs), hypothesis testing is initialized by finding the $\alpha^{\mathrm{th}}$ percentile from the null distribution. This critical value (T*) corresponds to the limit at which the null hypothesis can be rejected. Subsequently, the variances of windowed metrics (initial data) from all subjects were considered, that is, a matrix with dimensions regions×regions×subjects as described in Section 2.4, and the average across subjects was calculated in order to derive conclusions at the group level. These average variances were then compared with the previously obtained value of T*, thus resulting in a total of 78 comparisons. If an observed value was found to be greater than T*, the null hypothesis that the examined region pair yields stationary connectivity was rejected and it was concluded that these two brain areas exhibit statistically significant dFC.

### 2.9 Implementation details

All aforementioned methodologies were implemented in MATLAB^®^ (MathWorks^®^, Natick, MA) based on in house scripts as well as already available code. Specifically, implementation of the MTD metric was based on code provided by (Shine et al., 2015), while ICOV calculation was implemented through the *L1precisionBCD.m* function (Schmidt, 2006). Moreover, for the implementation of information based metrics, that is, MI and VI, the MIToolbox v.3.0.1 was utilized (Brown et al., 2012). Code snippets from Schneider and Neumaier, (2001) and Kugiumtzis and Tsimpiris, (2010) were employed for implementing the MVAR surrogate method, while the implementation of MVPR was based on the publicly available code at https://github.com/CommonClimate/common-climate/blob/master/phaseran.m (version 21/08/2011).

## 3 RESULTS

### 3.1 Effect of window size on windowed metric time‐series

**Figure 2:**
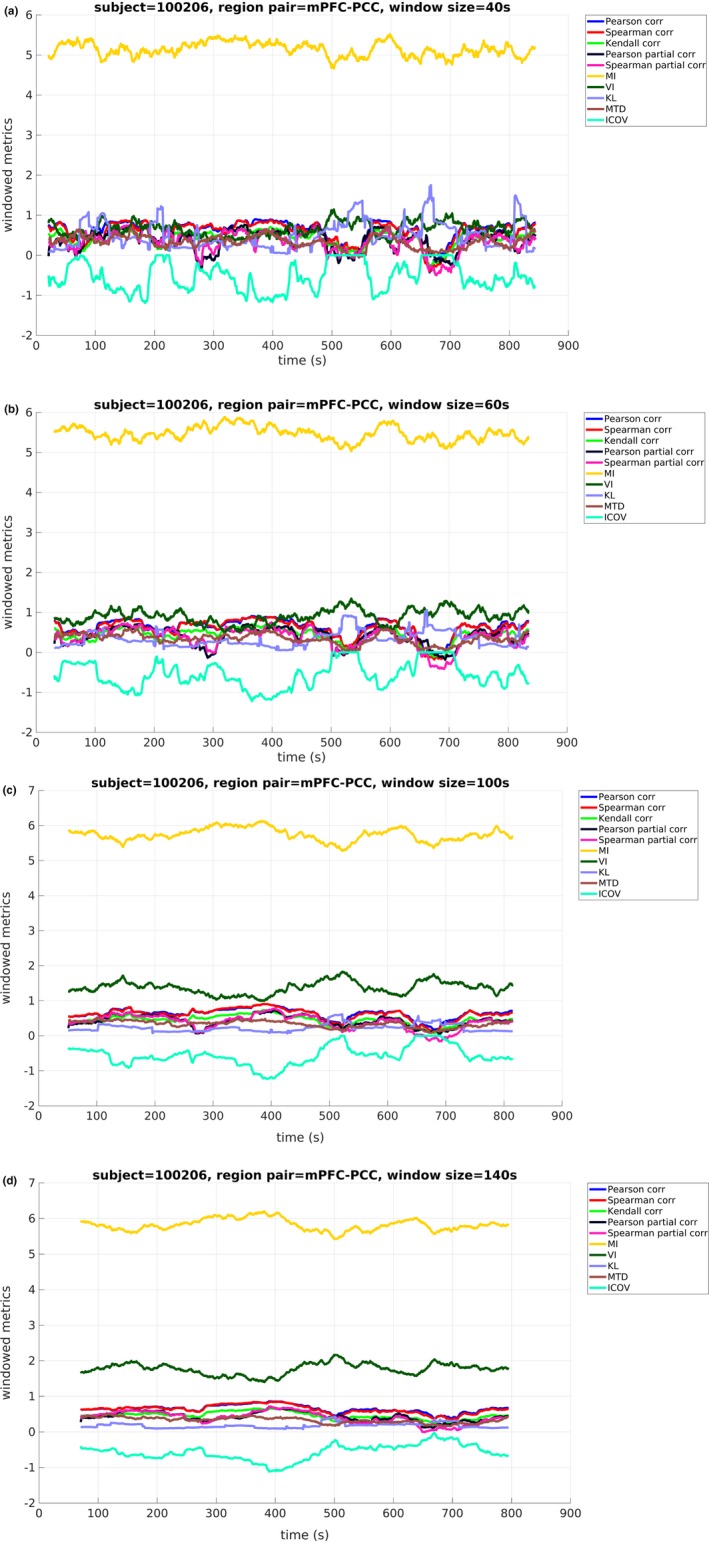
Windowed metrics between medial prefrontal cortex (mPFC) and posterior cingulate cortex (PCC) for all functional connectivity (FC) metrics and window lengths of (a) 40 s (b) 60 s (c) 100 s and (d) 140 s. A larger window size yielded in shorter windowed metric series converging to their mean value, different for each FC metric. *Metrics abbreviations:* MI: mutual information, VI: variation of information, KL: Kullback–Leibler divergence, MTD: multiplication of temporal derivatives, ICOV: Inverse Covariance

**Figure 3:**
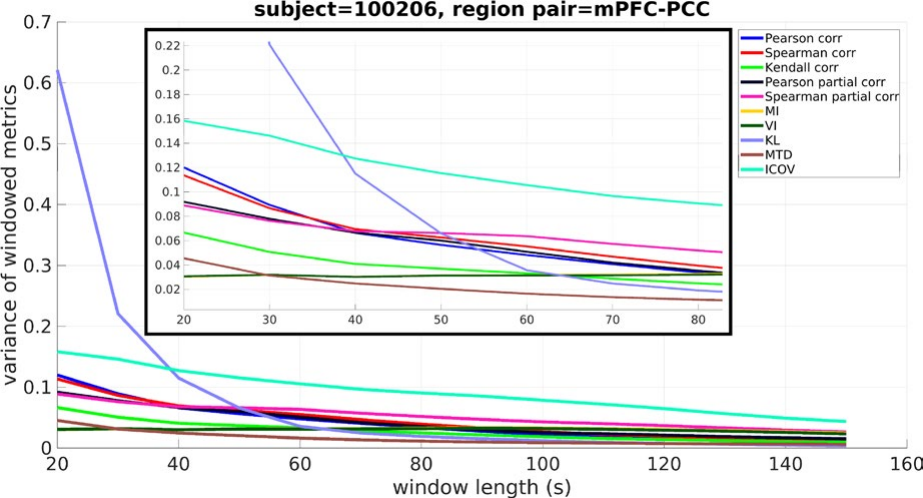
The variance of windowed metrics as a function of window size, for all examined functional connectivity metrics, sharply declines for window sizes between 20 and 60 s. For larger window sizes, variance gradually converges to zero. *Metrics abbreviations:* MI: mutual information, VI: variation of information, KL: Kullback–Leibler divergence, MTD: multiplication of temporal derivatives, ICOV: Inverse Covariance

To examine the effect of window size on the obtained windowed metric values, representative plots are presented for each metric and chosen window length (subject 100206—HCP nomenclature), indicating how the windowed metric values fluctuated over time. Specifically, Figure 2 shows the windowed metric variations during one scanning session between two core DMN regions (mPFC and PCC), for window sizes of 40, 60, 100, and 140 s. The windowed metrics in Figure 2 were plotted at the middle time point of each window size for easier visual comparison. Increasing window sizes resulted in shorter windowed metrics series, due to the lower total window number. Moreover, the obtained range of values for each FC metric decreased for increasing window size. Longer duration windows resulted in FC metric values that were gradually more concentrated around their mean value, whereby the latter was different for each metric. The same can be seen in Figure 3, where the variance of the windowed FC metrics is shown across all window lengths. In particular, Figure 3 illustrates the deviation for the full range of values and variance values between [0,0.2] (figure inset) for better resolution. Specifically, a decrease is observed in the variance values for the majority of FC metrics as the window size increases. This decline is steeper for window sizes between 20 and 60 s.

### 3.2 Reproducibility of dFC estimates

**Figure 4:**
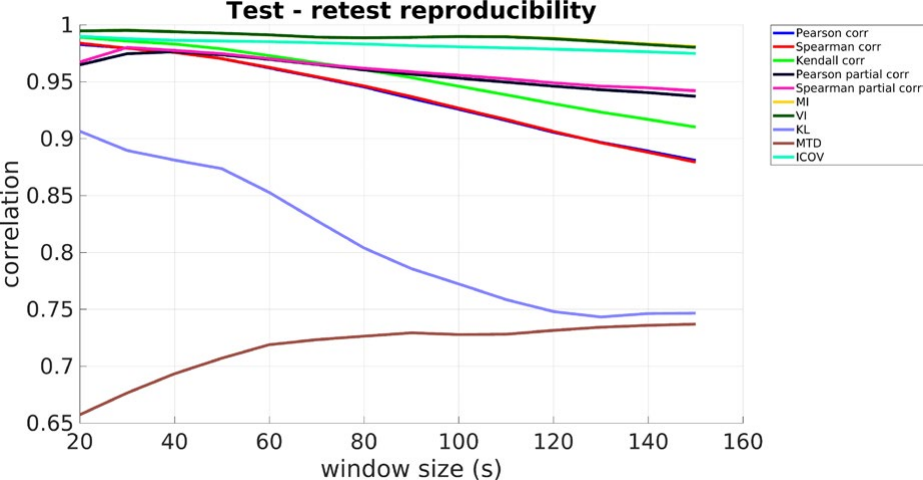
Test–retest reproducibility for all examined functional connectivity (FC) metrics and window sizes. MI and VI yielded the highest correlation of dynamic functional connectivity estimates between the two datasets, while KL and MTD seem to poorly perform in a test–retest approach, since the correlation was lower than the remaining FC metrics. *Metrics abbreviations:* MI: mutual information, VI: variation of information, KL: Kullback–Leibler divergence, MTD: multiplication of temporal derivatives, ICOV: Inverse Covariance

To assess the reproducibility of the examined FC metrics in the context of assessing dFC, the initial dataset of 100 subjects was divided into two disjoint groups each one consisting of 50 subjects (Section 2.1). To examine the degree of similarity, Pearson correlation was calculated between dFC estimates (variance of windowed metric time‐series) for all 78 ROI pairs of the two groups, for each FC metric and window size, after averaging over all subjects within each group. The corresponding results are illustrated in Figure 4. As can be seen, the majority of metrics yielded high test–retest reproducibility (correlation) of dFC estimates throughout the examined window size range (correlation values above 0.85). Some exceptions include the KL and MTD, whereby the former showed a steep decline to correlation values around 0.75 for window sizes up to 120 s. Moreover, the MTD yielded the lowest correlation values compared to all other metrics, although it increased with an increasing window size. Figure 4 suggests that MI and VI are the most reproducible metrics overall. Moreover, although these two metrics yielded higher correlation values for smaller window sizes, for example, 20–60 s, it should not be interpreted as a suitable window size range to use, as the estimation of dFC in these window lengths could suffer from a poor estimate of the respective PDFs due to the small number of data points inside each window.

### 3.3 Effect of FC metric and window size to the number of dynamic connections

The use of fluctuations in the windowed metrics (Section 3.1) as an indicative measure of dFC may be misleading as the calculated FC values are an estimation of the true FC (Hindriks et al., 2016). The results presented in Figures 2 and 3 provide some insights regarding the effect of window size on the variance of the windowed metrics, while from Figure 4 it is possible to export conclusions regarding the reproducibility of dFC estimates from each FC metric. However, these illustrations cannot lead to an answer to the question: “can the examined FC metric identify dynamically connected regions in rs‐fMRI?” Moreover, the second scope of the present study is to explore the effect of window size on resting‐state dFC analyses. Even though Figure 3 suggests that the variance of the windowed metrics remains relatively stable for window sizes larger than 60 s and Figure 4 suggests that the test–retest correlation of MI and VI was higher for window sizes [20s,60s], they cannot be directly used to identify a suitable value or range of values for the window length. Therefore, the statistical analysis framework described in Sections 2.5.8, 2.5.9 was employed to assess the presence of dFC ($H_{0}$ is rejected) between all possible region pairs for all metrics and window sizes, at a significance level of 0.05. To control for family‐wise error rate, the Bonferroni correction was employed (Hindriks et al., 2016). Moreover, a single null distribution for all pairs was utilized by aggregating individual distributions, to obtain a distribution with a large number of samples (250×78=19500), following Zalesky et al. (2014).

**Figure 5:**
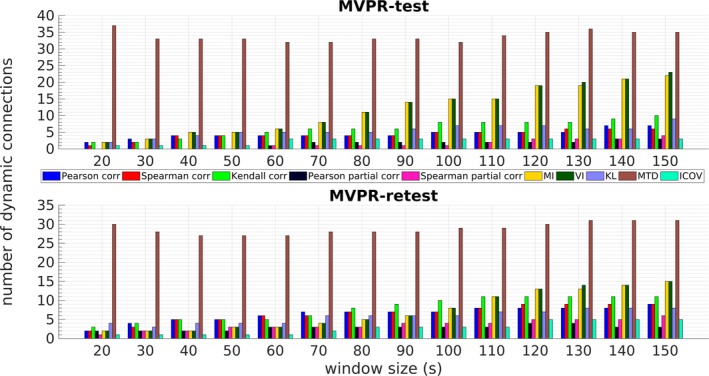
Number of dynamically connected regions for all functional connectivity metrics and window sizes with the multivariate phase randomization (MVPR) approach, using test (upper) and retest (lower) datasets. In general, an increasing window size yielded in more dynamically connected regions of interest for all metrics compared to a shorter one, except MTD metric. *Metric abbreviations:* MI: mutual information, VI: variation of information, KL: Kullback–Leibler divergence, MTD: multiplication of temporal derivatives, ICOV: Inverse Covariance

The corresponding number of dynamically connected region pairs using the MVPR approach is illustrated in Figure 5 for both the test and retest datasets. The corresponding results from the MVAR approach are presented in Section S3 in the Supporting information (Figure S3). A general remark is that longer windows generally yielded a larger number of region pairs exhibiting dFC, except for the case of MTD for both the test and retest datasets. In this case, the number of dynamically connected regions was significantly higher (≥25) compared to the remaining metrics. The MI and VI yielded more than 10 dynamically connected region pairs for window sizes larger than 80 and 110 s for the test–retest datasets, respectively.

### 3.4 Identifying dynamic connections with the Posterior Cingulate Cortex

The number of dynamically connected regions should not be interpreted as increased statistical power for each metric and window size, due to the absence of a ground truth (Sadia Shakil et al., 2016). In Section 3.2, it was suggested from Figure 4, that MI and VI yielded high correlation values with respect to dFC estimates between the test and retest groups, without considering which specific region pairs corresponded to statistically significant dFC.

To identify which metric and window size values are most suitable to use in dFC analyses, results from the study of Chang and Glover (2010) were employed. Specifically, in Chang and Glover (2010) a seed in the PCC was utilized for examining the presence of dynamic connections with both correlated (mPFC, L/R–IP) and anticorrelated (L/R insula, L/R dorsolateral prefrontal cortex, L/R supramarginal gyrus) brain regions. Analysis based on the wavelet transform concluded that coherence and phase coupling between these regions and the PCC was variable in time and frequency, providing evidence of dFC (Chang & Glover, 2010). In Table 2, results are shown for the MVPR method, (the corresponding ones from the MVAR technique are illustrated in Table S1 in the Supporting information), focusing on the PCC–mPFC, PCC–R‐IP, and PCC–L‐IP pairs, which were commonly identified in Chang and Glover (2010) and in the present study. As can be seen, MI and VI identified some of the aforementioned pairs (i.e., PCC–R‐IP) as dynamically connected using window sizes as small as 40 s. However, to characterize all the aforementioned dynamic connections, a window size of 120 s was found to be necessary for both datasets, except for the case of PCC–L‐IP in Dataset B (retest), where the corresponding FC was labeled as stationary. Based on these results, a window size of 120 s can be deemed as adequate, since it yielded all these ROI pairs as being dynamically connected. In the case of MVAR approach (Table S1 in the Supporting information), MI and VI also yielded good performance with a slightly larger window size (150 s). When utilizing MVPR and MTD, all the aforementioned pairs were identified as exhibiting dFC for window sizes larger than 20 s. Furthermore, some of the identified dynamically connected pairs involved the Cerebellum, which has been previously characterized as being the least dynamic (Zalesky et al., 2014), suggesting that this metric may be overly sensitive with respect to dFC detection. Finally, the remaining metrics, identified only one (e.g., ICOV) or no (e.g., Pearson linear correlation) dynamic connections, suggesting that these metrics are less sensitive with regard to detecting dFC.

**Table 2:** Connections with the PCC identified as dynamic using the sliding window approach for all examined FC metrics, using the MVPR approach

MVPR						
Metric	PCC–mPFC		PCC–R‐IP		PCC–L‐IP	
	Dataset A	Dataset B	Dataset A	Dataset B	Dataset A	Dataset B
Pearson linear correlation	–	–	–	–	–	–
Pearson partial linear correlation	–	–	–	–	–	–
Inverse Covariance matrix	≥20 s	≥20 s	–	–	–	–
Spearman rank correlation	–	–	–	≥120 s	–	–
Spearman partial rank correlation	–	≥120 s	–	–	–	–
Kendall correlation	–	–	–	≥70 s	≥140 s	–
Mutual Information	≥70 s	≥120 s	≥40 s	≥90 s	≥100 s	–
Variation of Information	≥70 s	≥120 s	≥40 s	≥90 s	≥90 s	–
Kullback–Leibler	–	–	–	–	–	–
Multiplication of Temporal Derivatives	≥20 s	≥20 s	≥20 s	≥20 s	≥20 s	≥20 s

Note

L‐IP: Left Inferior Parietal; mPFC: medial prefrontal cortex; MVPR: multivariate phase randomization; PCC: posterior cingulate cortex; R‐IP: right inferior parietal.

### 3.5 Identifying dynamic connections between all region pairs

To further assess the effect of different FC metrics and window size values on the results, we provide a detailed list of the dynamically connected regions in Table 3, using the MVPR technique. The corresponding results from the MVAR approach can be found in Table S2 in the Supporting information. The third and fifth column in Table 3 list the minimum window size which resulted in rejecting the null hypothesis for each FC metric and region pair. In all cases, the respective region pairs were found to be dynamically connected for all window sizes larger than these minimum values, except when an indication “†” is provided, for example, the pair mPFC–L‐IP, in the retest group, was identified as being dynamically connected using Pearson partial correlation and window sizes [120s,130s]. In the latter case, the “window size” column reports the range of window length, whereby the corresponding regions yielded dFC. The results obtained by employing the MTD metric were different in terms of the identified dynamic connections; that is, if a pair was identified as dynamically connected for a particular window size, the same pair was identified as exhibiting stationary FC for a larger window size. This was also shown in Figure 5, where the number of dynamically connected regions identified with MTD did not increase with an increasing window size. Therefore, all dynamic connections of MTD are presented in Figures S4 and S5 in the Supporting information, for all window sizes and the test–retest datasets respectively. All metrics identified region pairs among the frontal lobe, posterior cingulate cortex as well as the inferior parietal lobes and precuneus as exhibiting dynamic associations during the scanning session, which are generally in line with previously reported results (Chang & Glover, 2010; Zalesky et al., 2014). However, this delineation of pairs exhibiting dFC occurs at different window sizes for each FC metric.

**Table 3:** Dynamically connected region pairs identified using the sliding window and MVPR approach for different FC metrics and window sizes. In all cases, dFC between regions listed in the second and fourth column were detected for all window sizes larger than the value reported in the third and fifth column, respectively, unless indicated with “†.” In the latter case the “window size” column reports the range of window length whereby the corresponding regions yielded dFC

Metric	Dataset A		Dataset B	
	Dynamically connected regions	Window size (s)	Dynamically connected regions	Window size (s)
Pearson linear correlation	mPFC–R‐IP	≥20	mPFC–R‐IP	≥20
	mPFC–L‐IP	≥40	mPFC–L‐IP	≥30
	mPFC–Prec	≥20	mPFC–Prec	≥20
	PCC–Prec	≥100	PCC–Prec	≥60
	L‐IP–R‐MFG	≥140	L‐IP–R‐MFG	≥110
	L‐IP(2)–Prec	≥30	L‐IP(2)–Prec	≥30
	R‐MFG–Prec	≥140	R‐MFG–Prec	≥150
	–	–	mPFC–L‐MFG	≥40
	–	–	R‐IP–L‐IP(2)	≥70
Pearson linear partial correlation	mPFC–R‐IP	≥70	mPFC–R‐IP	≥20
	mPFC–Prec	≥60	mPFC–Prec	≥20
	PCC–Prec	≥140	PCC–Prec	≥60
	–	–	mPFC–L‐IP (†)	[120, 130]
Inverse Covariance	mPFC–PCC	≥20	mPFC–PCC	≥20
	mPFC–R‐MFG	≥60	mPFC–R‐MFG	≥70
	L‐IP–Prec	≥60	L‐IP–Prec	≥120
	–	–	R‐IP–Prec	≥80
	–	–	R‐IP–R‐MFG	≥120
Spearman rank correlation	mPFC–R‐IP	≥20	mPFC–R‐IP	≥20
	mPFC–L‐IP	≥40	mPFC–L‐IP	≥30
	mPFC–Prec	≥30	mPFC–Prec	≥20
	PCC–Prec	≥100	PCC–Prec	≥60
	L‐IP–R‐MFG	≥130	L‐IP–R‐MFG	≥110
	L‐IP(2)–Prec	≥40	L‐IP(2)–Prec	≥40
	–	–	R‐IP–L‐IP(2)	≥80
	–	–	mPFC–L‐MFG	≥40
	–	–	PCC–R‐IP	≥120
Spearman rank partial correlation	mPFC–R‐IP	≥110	mPFC–R‐IP	≥30
	mPFC–Prec	≥60	mPFC–Prec	≥20
	PCC–Prec	≥150	PCC–Prec	≥50
	mPFC–L‐MFG	≥120	mPFC–R‐MFG	≥150
	–	–	mPFC–PCC	≥120
	–	–	mPFC–L‐IP	≥90
Kendall correlation	mPFC–R‐IP	≥20	mPFC–R‐IP	≥20
	mPFC–L‐IP	≥40	mPFC–L‐IP	≥20
	mPFC–Prec	≥20	mPFC–Prec	≥20
	mPFC–L‐MFG	≥60	mPFC–L‐MFG	≥30
	PCC–Prec	≥70	PCC–Prec	≥40
	PCC–L‐MFG	≥150	PCC–L‐MFG	≥80
	L‐IP–R‐MFG	≥100	L‐IP–R‐MFG	≥100
	L‐IP(2)–Prec	≥50	L‐IP(2)–Prec	≥80
	R‐MFG–Prec	≥100	R‐MFG–Prec	≥110
	PCC–L‐IP	≥140	PCC–R‐IP	≥70
	–	–	R‐IP–L‐IP(2)	≥90
Mutual information	mPFC–PCC	≥70	mPFC–PCC	≥120
	mPFC–R‐IP	≥30	mPFC–R‐IP	≥80
	mPFC–L‐IP	≥80	mPFC–L‐IP	≥120
	mPFC–R‐MFG	≥90	mPFC–R‐MFG	≥140
	mPFC–Prec	≥60	mPFC–Prec	≥70
	PCC–R‐IP	≥40	PCC–R‐IP	≥90
	PCC–Prec	≥80	PCC–Prec	≥100
	R‐IP–L‐IP	≥20	R‐IP–L‐IP	≥20
	R‐IP–R‐MFG	≥70	R‐IP–R‐MFG	≥100
	R‐IP–Prec	≥20	R‐IP–Prec	≥20
	R‐IP–L‐MFG	≥80	R‐IP–L‐MFG	≥110
	L‐IP–Prec	≥40	L‐IP–Prec	≥50
	L‐IP–L‐MFG	≥120	L‐IP–L‐MFG	≥150
	R‐MFG–Prec	≥90	R‐MFG–Prec	≥110
	Prec–L‐MFG	≥90	Prec–L‐MFG	≥110
	L‐IP–R‐MFG	≥120	–	–
	L‐IP(2)–Prec	≥140	–	–
	L‐Hipp–R‐IP	≥150	–	–
	L‐Hipp–Prec	≥120	–	–
	mPFC–L‐MFG	≥120	–	–
	PCC–L‐IP	≥100	–	–
	PCC–R‐MFG	≥140	–	–
Variation of information	mPFC–PCC	≥70	mPFC–PCC	≥120
	mPFC–R‐IP	≥30	mPFC–R‐IP	≥80
	mPFC–L‐IP	≥80	mPFC–L‐IP	≥120
	mPFC–R‐MFG	≥100	mPFC–R‐MFG	≥130
	mPFC–Prec	≥60	mPFC–Prec	≥70
	PCC–R‐IP	≥40	PCC–R‐IP	≥90
	PCC–Prec	≥80	PCC–Prec	≥100
	R‐IP–L‐IP	≥20	R‐IP–L‐IP	≥20
	R‐IP–R‐MFG	≥70	R‐IP–R‐MFG	≥100
	R‐IP–Prec	≥20	R‐IP–Prec	≥20
	R‐IP–L‐MFG	≥80	R‐IP–L‐MFG	≥110
	L‐IP–Prec	≥40	L‐IP–Prec	≥50
	L‐IP–L‐MFG	≥120	L‐IP–L‐MFG	≥150
	R‐MFG–Prec	≥90	R‐MFG–Prec	≥110
	Prec–L‐MFG	≥90	Prec–L‐MFG	≥110
	L‐IP–R‐MFG	≥120	–	–
	PCC–L‐MFG	≥150	–	–
	L‐IP(2)–Prec	≥140	–	–
	L‐Hipp–R‐IP	≥150	–	–
	L‐Hipp–Prec	≥120	–	–
	mPFC–L‐MFG	≥120	–	–
	PCC–L‐IP	≥90	–	–
	PCC–R‐MFG	≥130	–	–
Kullback–Leibler divergence	mPFC–R‐IP	≥20	mPFC–R‐IP	≥20
	mPFC–L‐IP	≥50	mPFC–L‐IP	≥40
	mPFC–Prec	≥40	mPFC–Prec	≥20
	mPFC–L‐MFG	≥100	mPFC–L‐MFG	≥70
	R‐IP–L‐IP(2) (†)	[30, 120]	R‐IP–L‐IP(2) (†)	20, [70, 150]
	L‐IP(2)–Prec	≥20	L‐IP(2)–Prec	≥20
	L‐IP–L‐IP(2)	≥150	PCC–Prec	≥110
	Cer–mPFC	≥150	PCC–L‐MFG	≥130
	L‐Hipp–Prec	≥150	–	–
	R‐IP–ACG	≥90	–	–
Multiplication of Temporal Derivatives	[Link]	[Link]	[Link]	[Link]

Note

ACG: anterior cingulate gyrus; Cer: cerebellum; dFC: dynamic functional connectivity; FC: functional connectivity; L‐Hipp: left hippocampus; L‐IP: left inferior parietal; L‐MFG: left middle frontal gyrus; mPFC: medial prefrontal cortex; MVPR: multivariate phase randomization; PCC: posterior cingulate cortex; Prec: Precuneus; R‐Hipp: right hippocampus; R‐IP: right inferior parietal; R‐MFG: right middle frontal gyrus; ROI: region of interest; Thal: Thalamus.

Please refer to Supporting information (Section S4) for all dynamically connected ROI pairs for each window size.

**Figure 6:**
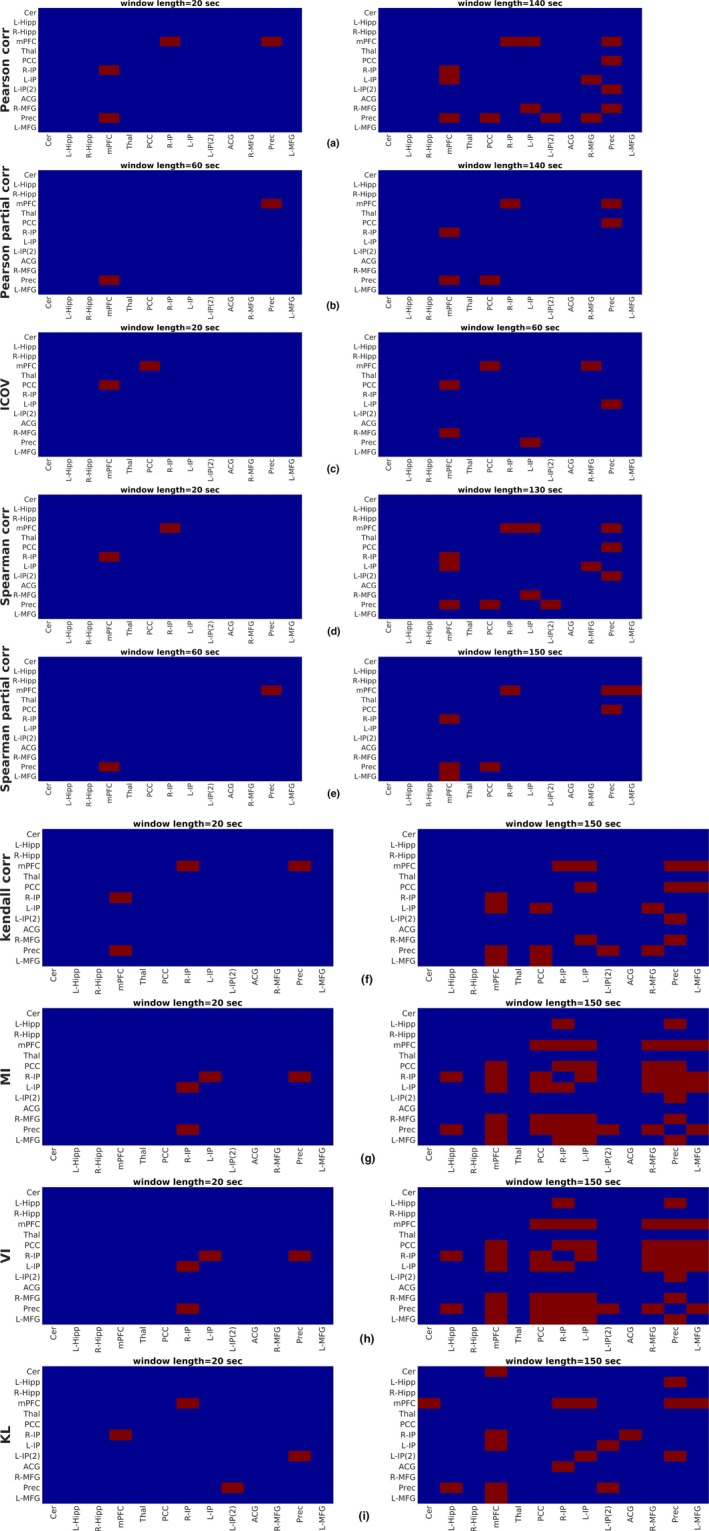
Statistical inference on dynamic functional connectivity utilizing the test set with (a) Pearson linear correlation, (b) Pearson partial correlation, (c) ICOV, (d) Spearman rank correlation, (e) Spearman partial correlation, (f) Kendall correlation, (g) MI, (h) VI, and (i) KL divergence. Left panels correspond to the minimum window size whereby dynamically connected regions were identified, while right panels correspond to a window size above which no dynamic connections were highlighted. Results of hypothesis testing using the MTD are explicitly shown in Figure [Link], [Link] of Supporting information, for all window sizes. *Metrics abbreviations:* ICOV: Inverse Covariance, MI: mutual information, VI: variation of information, KL: Kullback–Leibler divergence, MTD: Multiplication of Temporal Derivatives. *Regions abbreviations:* Cer: cerebellum, L‐Hipp: left hippocampus, R‐Hipp: right hippocampus, mPFC: medial prefrontal cortex, Thal: thalamus, PCC: posterior cingulate cortex, R‐IP: right inferior parietal, L‐IP: left inferior parietal, L‐IP(2): left inferior parietal–2, ACG: anterior cingulate gyrus, R‐MFG: right middle frontal gyrus, Prec: precuneus, L‐MFG: left middle frontal gyrus

**Figure 7:**
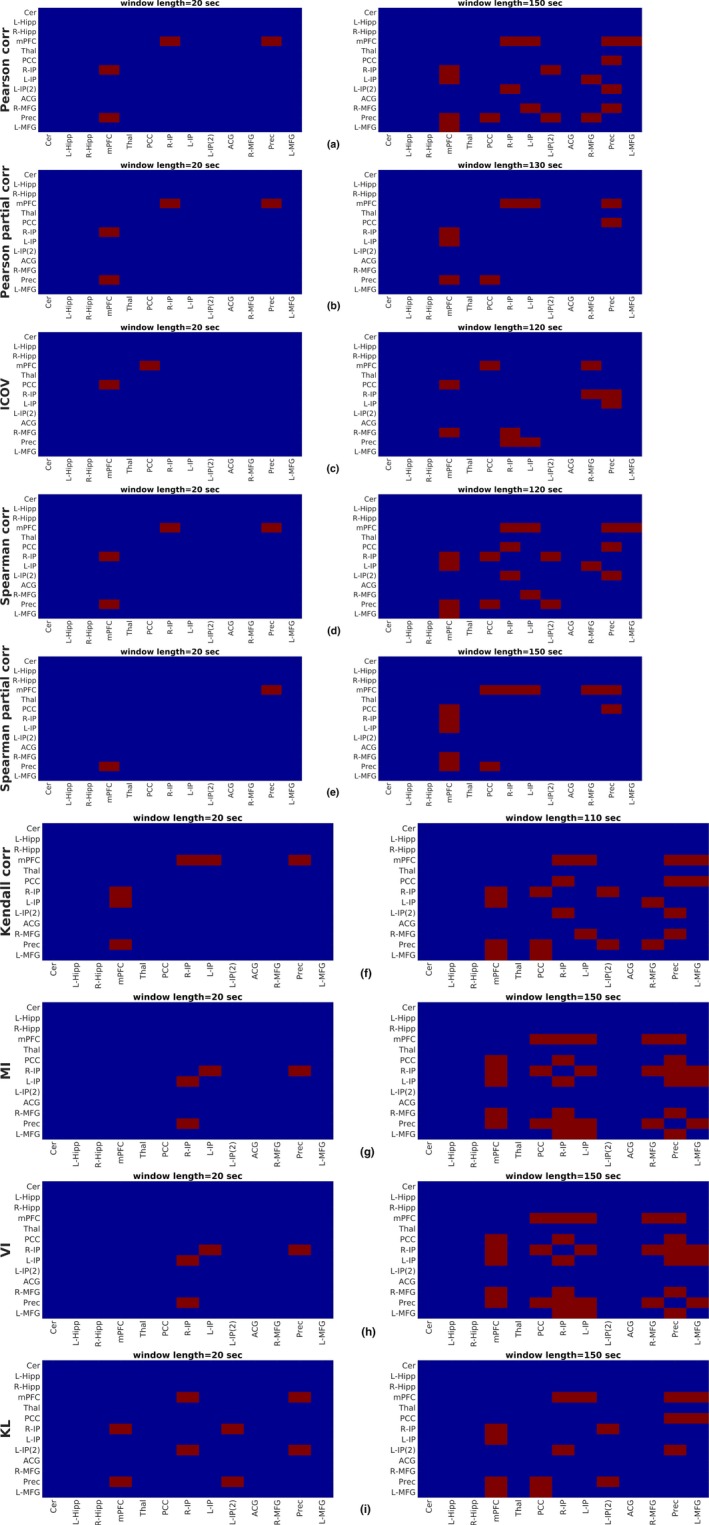
Statistical inference on dynamic functional connectivity utilizing the retest set with (a) Pearson linear correlation, (b) Pearson partial correlation, (c) ICOV, (d) Spearman rank correlation, (e) Spearman partial correlation, (f) Kendall correlation, (g) MI, (h) VI, and (i) KL divergence. Left panels correspond to the minimum window size whereby dynamically connected regions were identified, while right panels correspond to a window size above which no dynamic connections were highlighted. Results of hypothesis testing using the MTD are explicitly shown in Figure [Link], [Link] of Supporting information, for all window sizes. *Metrics abbreviations:* ICOV: Inverse Covariance, MI: mutual information, VI: variation of information, KL: Kullback–Leibler divergence, MTD: Multiplication of Temporal Derivatives. *Regions abbreviations:* Cer: cerebellum, L‐Hipp: left hippocampus, R‐Hipp: right hippocampus, mPFC: medial prefrontal cortex, Thal: thalamus, PCC: posterior cingulate cortex, R‐IP: right inferior parietal, L‐IP: left inferior parietal, L‐IP(2): left inferior parietal–2, ACG: anterior cingulate gyrus, R‐MFG: right middle frontal gyrus, Prec: precuneus, L‐MFG: left middle frontal gyrus

The results of Table 3 are also visualized in Figures 6 and 7 in the form of 13×13 matrices, for the test and retest datasets, respectively. Red color denotes the pair of regions exhibiting dFC and blue color indicates the region pairs which were not found to be dynamically connected ($H_{0}$ could not be rejected). The left panels of Figures 6 and 7 present the results of hypothesis testing for the minimum window size for which any dynamic connections were detected, that is, 20 s for all metrics except Pearson and Spearman partial correlation (Figure 6b,e), for which this size was found to be 60 s. In agreement to Table 3, smaller window sizes yielded a lower number of dynamically connected regions. The right column of Figures 6 and 7 corresponds to the window size above which no additional dynamically connected regions were identified (third and fifth column of Table 3).

The results of hypothesis testing as presented in Figures 6 and 7, display a binary outcome: $H_{0}$ rejected or accepted. Therefore, they do not specify which connections are more dynamic in comparison to others. To this end, we show the averaged (over all subjects within test and retest datasets) obtained variance of windowed metric values for all significant dynamic connections in Table 4, which illustrates in detail the dFC strength of significantly dynamic edges in descending order. Table 4 suggests that specific region pairs exhibit more pronounced dFC, suggesting that they exhibited higher dFC values (variance of windowed metric values—see also Section 2.8) compared to other pairs using the same FC metric – note that they should not be interpreted as isolated numbers. For instance, in Table 4, a dFC strength value of 0.0265 (mPFC–Prec pair in Dataset A) using Pearson linear correlation, compared to values from the same metric, for example, from 0.0245 (mPFC–L‐IP pair in Dataset A) to 0.0183 (R‐MFG–Prec pair in Dataset A), provides evidence of a stronger dFC for the mPFC–Prec pair relative to other pairs, for example, mPFC–L‐IP, using Pearson linear correlation.

**Table 4:** DFC strength of significant edges in descending order, using the MVPR approach

Metric–window size (s)	Dataset A		Dataset B	
	Dynamically connected regions	dFC strength	Dynamically connected regions	dFC strength
Pearson linear correlation—150	mPFC–Prec	0.0265	mPFC–Prec	0.0312
	mPFC–L‐IP	0.0245	mPFC–R‐IP	0.0304
	L‐IP(2)–Prec	0.0241	mPFC–L‐MFG	0.0255
	mPFC–R‐IP	0.0234	mPFC–L‐IP	0.0241
	PCC–Prec	0.0197	L‐IP(2)–Prec	0.0239
	L‐IP–R‐MFG	0.0183	R‐IP–L‐IP(2)	0.0228
	R‐MFG–Prec	0.0183	PCC–Prec	0.0223
	–	–	L‐IP–R‐MFG	0.0211
	–	–	R‐MFG–Prec	0.0202
Pearson linear partial correlation—140	mPFC–Prec	0.0171	mPFC–R‐IP	0.0174
	mPFC–R‐IP	0.0170	mPFC–Prec	0.0171
	PCC–Prec	0.0157	PCC–Prec	0.0161
Inverse Covariance—120	L‐IP–Prec	0.0358	mPFC–PCC	0.0359
	mPFC–PCC	0.0319	mPFC–R‐MFG	0.0346
	mPFC–R‐MFG	0.0313	R‐IP–Prec	0.0341
	–	–	R‐IP–R‐MFG	0.0309
	–	–	L‐IP–Prec	0.0307
Spearman rank correlation—130	mPFC–Prec	0.0297	mPFC–Prec	0.0335
	mPFC–L‐IP	0.0276	mPFC–R‐IP	0.0333
	L‐IP(2)–Prec	0.0273	mPFC–L‐MFG	0.0285
	mPFC–R‐IP	0.0258	mPFC–L‐IP	0.0266
	PCC–Prec	0.0226	L‐IP(2)–Prec	0.0258
	L‐IP–R‐MFG	0.0218	PCC–Prec	0.0253
	–	–	R‐IP–L‐IP(2)	0.0252
	–	–	L‐IP–R‐MFG	0.0241
	–	–	PCC–R‐IP	0.0234
Spearman rank partial correlation—150	mPFC–Prec	0.0155	mPFC–R‐IP	0.0155
	mPFC–R‐IP	0.0147	mPFC–Prec	0.0155
	mPFC–L‐MFG	0.0143	mPFC–PCC	0.0147
	PCC–Prec	0.0141	mPFC–L‐IP	0.0146
	–	–	PCC–Prec	0.0146
	–	–	mPFC–R‐MFG	0.0140
Kendall correlation—150	mPFC–Prec	0.0142	mPFC–Prec	0.0153
	mPFC–L‐IP	0.0129	mPFC–R‐IP	0.0149
	mPFC–R‐IP	0.0119	mPFC–L‐MFG	0.0129
	PCC–Prec	0.0118	PCC–Prec	0.0122
	L‐IP(2)–Prec	0.0114	mPFC–L‐IP	0.0118
	L‐IP–R‐MFG	0.0105	PCC–R‐IP	0.0113
	mPFC–L‐MFG	0.0099	R‐IP–L‐IP(2)	0.0107
	R‐MFG–Prec	0.0098	L‐IP–R‐MFG	0.0106
	PCC–L‐IP	0.0096	L‐IP(2)–Prec	0.0106
	PCC–L‐MFG	0.0096	R‐MFG–Prec	0.0104
	–	–	PCC–L‐MFG	0.0099
Mutual information—150	R‐IP–Prec	0.0582	R‐IP–Prec	0.0523
	L‐IP–Prec	0.0550	R‐IP–L‐IP	0.0478
	R‐IP–L‐IP	0.0501	L‐IP–Prec	0.0478
	mPFC–Prec	0.049	mPFC–Prec	0.0454
	PCC–Prec	0.0471	mPFC–R‐IP	0.0401
	R‐MFG–Prec	0.0458	R‐MFG–Prec	0.0394
	Prec–L‐MFG	0.0452	PCC–Prec	0.0388
	PCC–R‐IP	0.0437	Prec–L‐MFG	0.0386
	mPFC–R‐IP	0.0430	R‐IP–R‐MFG	0.0376
	R‐IP–R‐MFG	0.0423	mPFC–PCC	0.0372
	mPFC–PCC	0.0415	R‐IP–L‐MFG	0.0365
	mPFC–L‐IP	0.0401	mPFC–L‐IP	0.0363
	R‐IP–L‐MFG	0.0399	PCC–R‐IP	0.0363
	PCC–L‐IP	0.0396	mPFC–R‐MFG	0.0357
	mPFC–R‐MFG	0.0393	L‐IP–L‐MFG	0.0346
	L‐Hipp–Prec	0.0390	–	–
	L‐IP–R‐MFG	0.0372	–	–
	L‐IP–L‐MFG	0.0368	–	–
	mPFC–L‐MFG	0.0364	–	–
	PCC–R‐MFG	0.0359	–	–
	L‐IP(2)–Prec	0.0351	–	–
	L‐Hipp–R‐IP	0.0336	–	–
Variation of information—150	R‐IP–Prec	0.0568	R‐IP–Prec	0.0507
	L‐IP–Prec	0.0542	L‐IP–Prec	0.0469
	mPFC–Prec	0.0486	R‐IP–L‐IP	0.0465
	R‐IP–L‐IP	0.0486	mPFC–Prec	0.0448
	PCC–Prec	0.0460	mPFC–R‐IP	0.0393
	R‐MFG–Prec	0.0452	R‐MFG–Prec	0.0390
	Prec–L‐MFG	0.0444	PCC–Prec	0.0385
	PCC–R‐IP	0.0430	Prec–L‐MFG	0.0379
	mPFC–R‐IP	0.0424	R‐IP–R‐MFG	0.0373
	mPFC–PCC	0.0411	mPFC–PCC	0.0366
	R‐IP–R‐MFG	0.0411	R‐IP–L‐MFG	0.0360
	mPFC–L‐IP	0.0394	PCC–R‐IP	0.0358
	R‐IP–L‐MFG	0.0394	mPFC–L‐IP	0.0357
	PCC–L‐IP	0.0393	mPFC–R‐MFG	0.0355
	L‐Hipp–Prec	0.0386	L‐IP–L‐MFG	0.0339
	mPFC–R‐MFG	0.0386	–	–
	L‐IP–R‐MFG	0.0366	–	–
	L‐IP–L‐MFG	0.0360	–	–
	mPFC–L‐MFG	0.0358	–	–
	PCC–R‐MFG	0.0357	–	–
	L‐IP(2)–Prec	0.0347	–	–
	L‐Hipp–R‐IP	0.0330	–	–
	PCC–L‐MFG	0.0330	–	–
Kullback–Leibler divergence—120	mPFC–R‐IP	0.0115	mPFC–R‐IP	0.0134
	mPFC–L‐IP	0.0105	mPFC–Prec	0.0120
	mPFC–Prec	0.0100	mPFC–L‐IP	0.0110
	L‐IP(2)–Prec	0.0097	mPFC–L‐MFG	0.0108
	R‐IP–ACG	0.0091	L‐IP(2)–Prec	0.0108
	mPFC–L‐MFG	0.0089	R‐IP–L‐IP(2)	0.0104
	Cer–mPFC	0.0086	PCC–L‐MFG	0.0096
	L‐IP–L‐IP(2)	0.0085	PCC–Prec	0.0095
	L‐Hipp–Prec	0.0084	–	–
Multiplication of Temporal Derivatives—150	PCC–R‐IP	0.0329	mPFC–R‐MFG	0.0434
	R‐IP–Prec	0.0290	mPFC–L‐MFG	0.0313
	mPFC–R‐MFG	0.0286	PCC–Prec	0.0206
	PCC–Prec	0.0264	R‐IP–L‐IP	0.0201
	mPFC–R‐IP	0.0252	mPFC–Prec	0.0196
	mPFC–Prec	0.0212	mPFC–PCC	0.0192
	mPFC–PCC	0.0205	R‐MFG–L‐MFG	0.0188
	mPFC–L‐MFG	0.0183	mPFC–R‐IP	0.0177
	R‐IP–L‐MFG	0.0179	PCC–L‐MFG	0.0177
	R‐IP–L‐IP	0.0177	mPFC–L‐IP	0.0174
	R‐IP–L‐IP(2)	0.0156	R‐IP–Prec	0.0154
	R‐IP–R‐MFG	0.0153	R‐IP–R‐MFG	0.0147
	mPFC–L‐IP	0.0152	PCC–R‐IP	0.0146
	Prec–L‐MFG	0.0152	PCC–L‐IP	0.0144
	PCC–L‐MFG	0.0150	PCC–R‐MFG	0.0131
	PCC–R‐MFG	0.0136	R‐MFG–Prec	0.0129
	R‐MFG–Prec	0.0135	L‐IP–Prec	0.0127
	PCC–L‐IP	0.0127	L‐Hipp–Prec	0.0115
	L‐IP–Prec	0.0126	R‐Hipp–Thal	0.0115
	Cer–R‐IP	0.0124	mPFC–Thal	0.0114
	R‐MFG–L‐MFG	0.0123	Prec–L‐MFG	0.0114
	Cer–PCC	0.0120	L‐IP–R‐MFG	0.0112
	L‐IP(2)–Prec	0.0117	L‐IP–L‐IP(2)	0.0108
	L‐Hipp–PCC	0.0116	L‐Hipp–R‐Hipp	0.0106
	L‐IP–L‐IP(2)	0.0113	Cer–mPFC	0.0102
	PCC–L‐IP(2)	0.0111	R‐Hipp–Prec	0.0102
	L‐IP–R‐MFG	0.0104	L‐Hipp–mPFC	0.0099
	Cer–Prec	0.0102	mPFC–L‐IP(2)	0.0099
	L‐Hipp–Prec	0.0098	Cer–PCC	0.0096
	mPFC–L‐IP(2)	0.0098	R‐IP–L‐IP(2)	0.0096
	L‐Hipp–R‐Hipp	0.0093	L‐IP–L‐MFG	0.0096
	L‐Hipp–R‐IP	0.0091	–	–
	PCC–ACG	0.0087	–	–
	L‐IP–L‐MFG	0.0086	–	–
	R‐Hipp–Thal	0.0085	–	–

Note

ACG: anterior cingulate gyrus; Cer: cerebellum; dFC: dynamic functional connectivity; L‐Hipp: left hippocampus; L‐IP: left inferior parietal; L‐MFG: left middle frontal gyrus; mPFC: medial prefrontal cortex; MVPR: multivariate phase randomization; PCC: posterior cingulate cortex; Prec: Precuneus; R‐Hipp: right hippocampus; R‐IP: right inferior parietal; R‐MFG: right middle frontal gyrus; ROI: region of interest; Thal: Thalamus.

Region pairs exhibiting more pronounced dFC include connections of mPFC with Prec, R‐IP, L‐IP, as well as connections between PCC, R‐IP, L‐IP, and Prec. Particularly, dynamic connections between mPFC and Prec, R‐IP, L‐IP were the most prominent and were identified using the majority of FC metrics (Pearson full and partial correlation, Spearman full and partial correlation, Kendall correlation, KL divergence, ICOV). On the other hand, connections between the R‐IP/L‐IP and Prec were more pronounced in the case of MI and VI, while dynamic connections with the mPFC were weaker. These differences in dFC strength could be partly attributed to the use of different FC metrics for quantifying correlations between regions. Finally, it is only meaningful to compare dFC strength values for each FC metric. For instance, one cannot directly compare a dFC strength value of 0.0265 (mPFC–Prec pair in Dataset A), using Pearson linear correlation to the dFC strength value of 0.0142 (mPFC–Prec pair in Dataset A), using Kendall correlation, due to the different nature of these metrics. However, it can be concluded that, using two different metrics, the pair mPFC–Prec was sorted first among all pairs, suggesting that it is a strongly dynamic connection in the resting human brain. Moreover, results in Table 4 also suggest that FC metrics reproduced previous results regarding the most dynamically connected regions, since regions of the frontal and inferior parietal lobes were highlighted among those having the highest dFC strength (Zalesky et al., 2014). Specifically, in the case of MI and VI, the bilateral parietal lobes were found to be involved in the most dynamic connections, a result that is in agreement to (Zalesky et al., 2014).

## 4 DISCUSSION

### 4.1 Overview of the current study

The present study rigorously examined the sliding window methodology for assessing dFC in the DMN using rs‐fMRI data focusing on: (a) the effect of window size and (b) the effect of FC metric. To this end, a total of 14 window sizes between 20 and 150 s were employed, along with 10 FC metrics, some of which, to our knowledge, have not been used in previous rs‐fMRI studies. To assess the presence of dFC, surrogate data based on the MVPR and MVAR approaches were used to generate a suitable null hypothesis (dFC absence), focusing on the MVPR, as it better preserved properties of the initial data (Section 2.3 and Section S2 of Supporting information). The obtained results suggest that small window sizes (e.g., from 20 to 50 s) yielded relatively few dynamically connected regions, while longer windows yielded additional dynamic connections (Figures 5, 6, 7 and Table 2). Finally, MI and VI were found to yield the most reproducible dFC estimates (Figure 4) compared to all other FC metrics, identifying at the same time, dynamic connections that have been previously reported in the literature (Chang & Glover, 2010; Zalesky et al., 2014), using a window size larger than 120s (Table 2).

### 4.2 Previous work and comparison to the present study

In a recent study, Shakil et al. (2016) investigated the effect of different sliding window parameters, that is, window size, step and type on the assessment of dFC and the detection of brain states. The authors employed simulated resting‐state networks created by segmenting real BOLD responses at predefined time points and mixing the resulting time‐series to form an experimental setting where the transitions from one brain state to the other were known. Their results suggested that detecting brain state transitions was greatly affected by the chosen window size and step. Specifically, window sizes close to the duration of each state and small window offsets resulted in accurate detection of state changes in the simulated networks (Shakil et al., 2016).

In the present study, we sought to answer similar questions using experimental data instead of simulated BOLD time‐series, analyzing a total of 100 high quality rs‐fMRI data, from the HCP project (Smith, Beckmann et al., 2013) and dividing them into two separate groups of 50 subjects each, for facilitating a test–retest validation scheme. The effect of different sizes and metrics on dFC assessment was investigated. One important difference to the simulation study of Shakil et al. (2016) is there is no ground truth; therefore, we also compared our results to results reported in the literature. With regard to the effect of window size, it was found that an increasing window size resulted in windowed metric series that gradually converged toward their mean value, which was different for each metric (Figure 2). Similar observations were reported in Chang and Glover (2010), where the authors, additionally to wavelet analysis, utilized the sliding window method with Pearson linear correlation evaluated in windows of 2 and 4 min and in Hutchison, Womelsdorf, Gati et al. (2013). In the latter study, the above mentioned remarks were observed using Pearson linear correlation and window sizes of 30, 60, 120, and 240 s, for two datasets, that is, awake human and anesthetized macaques (Hutchison, Womelsdorf, Gati et al., 2013). However, this did not result in a larger number of dynamic connections for shorter windows; on the contrary, for all examined FC metrics, except MTD, a larger number of dynamically connected regions were identified for longer windows (Table 3 and Figure 5). For some FC metrics, the number of dynamic connections stabilized above a minimum window length value that was both region‐ and metric‐specific (Figures 6 & 7 and Table 3).

### 4.3 Number of dynamically connected region pairs

One important observation from the present study is that an increasing window size for almost all examined metrics yielded a larger number of dynamically connected region pairs for both MVPR (Figure 5) and MVAR (Figure S3 of Supporting information), except for MTD combined with MVPR. This can be justified by the fact that larger window sizes are able to capture slower fluctuations at a greater extent compared to shorter windows, whereby only faster oscillations can be captured, suggesting that low frequency components are more important for assessing dFC from rs‐fMRI compared to high frequency FC fluctuations (Achard, Salvador, Whitcher, Suckling, & Bullmore, 2006; Biswal, Zerrin Yetkin, Haughton, & Hyde, 1995; Salvador et al., 2005; Zou et al., 2008). In line with this, in Chang and Glover (2010), it was shown that regions strongly correlated to the PCC (mPFC, L/R–IP) exhibited dFC at a peak frequency of f≈0.016Hz, further supporting the remark of dominant low frequency components in the context of assessing dFC.

The segregation of the examined region pairs to dynamic and nondynamic also suggests that the DMN network may be functionally divided into multiple subsystems (Andrews‐Hanna et al., 2010; Andrews‐Hanna, Smallwood, & Spreng, 2014). The employed methodology considered the DMN as a single entity using a 13‐variable model for constructing surrogate data and aggregating all 78 individual null distributions to a single one. However, recent work proposed that this approach (considering the DMN as a single entity) may be somewhat simplistic, suggesting that the DMN can be divided into three distinct subsystems: the medial temporal (hippocampus, parahippocampal cortex, retrosplenial cortex, posterior inferior parietal lobe, and ventromedial prefrontal cortex), the dorsal medial (dorsal medial prefrontal cortex, temporoparietal junction, lateral temporal cortex, and the temporal pole), and the midline core (anterior medial prefrontal cortex and posterior cingulate cortex) (Andrews‐Hanna et al., 2010). Specifically, in Andrews‐Hanna et al., (2010), this hypothesis was examined using a hierarchical clustering approach and it was suggested that the medial temporal and dorsal medial subsystems were both highly correlated with the midline core, signifying that the DMN may be organized in different subsystems. For a more comprehensive discussion please refer to Andrews‐Hanna et al., (2014). This subsystem organization may be reflected on the presence of differentially dynamic connections between different region pairs within the DMN. The current set of methods, that is, the formulated hypothesis testing, only allows the distinction between two conditions which are as follows: dFC absence or presence for a given confidence level and therefore, a similar to the above reported distinction (DMN divided into three subsystems) cannot be obtained. Future work should address this by separating the initial 13 regions to three groups and examining dFC within these subsystems.

MTD yielded a large number (≥25) of dynamically connected pairs (Figure 5) using window sizes as small as 20 s, involving connections with the PCC (Table 2), as well as connections with the Cerebellum (Section S4 of Supporting information), which has been previously characterized as being the least dynamic (Zalesky et al., 2014). These results suggest that MTD may be overly sensitive with respect to dFC detection. One possible reason is that, as MTD relies on derivatives, it may be affected more by fast fluctuations in FC metrics and may also tend to amplify them, resulting in the identification of dFC even when using short window sizes, whereby larger fluctuations in FC values cannot be captured. However, this seems to result in spurious dFC estimation, as implied by the obtained results (Figure 5; Table 2 and Section S4 of Supporting information).

### 4.4 Suitability of FC metrics and window sizes for identifying dFC

To better identify the metrics and window sizes that are best suited for rs‐fMRI dFC analyses, results from the study of Chang and Glover (2010) were employed for further comparison. Specifically, Chang and Glover (2010) utilized a seed in the PCC for examining the presence of dynamic connections with both correlated (mPFC, L/R–IP) and anticorrelated (L/R insula, L/R dorsolateral prefrontal cortex, and L/R supramarginal gyrus) brain regions. To assess the presence of dFC, Chang and Glover (2010) estimated the wavelet transform of each BOLD signal for each region separately, as well as the cross wavelet spectrum between each region pair. Individual and cross wavelet transforms were then combined to estimate WTC—a measure of correlation in the time‐frequency domain similar to Pearson correlation (Chang & Glover, 2010; Grinsted, Moore, & Jevrejeva, 2004). Subsequently, high coherence areas in WTC maps were highlighted to yield Time‐Averaged Coherence (TAC) curves, which displayed peaks at particular frequencies, for example, f=0.016Hz and f=0.03Hz, (Section 4.3) indicating that each examined region pair interacted at different timescales, thus providing evidence of dFC (Chang & Glover, 2010).

Table 2 illustrates the PCC correlated regions from the study of Chang and Glover (2010) and the window sizes for which they were identified as dynamically connected using all FC metrics. For instance, the pair PCC–R‐IP was identified as dynamically connected with MI and VI using a window size of 40s in the test dataset. Simultaneously, the remaining pairs (PCC–mPFC and PCC–L‐IP) were characterized as exhibiting stationary FC using a window size of 40 s, while in the study of Chang and Glover (2010), they were identified as being dynamically connected.

In another study, Zalesky et al. (2014) employed a whole‐brain approach based on the Automated Anatomical Labeling (AAL) atlas with 6,670 unique pairs along with the sliding window technique. Pearson linear correlation combined with an exponential window size of 60s was used for identifying a set of 19 regions characterized as “consistently dynamic,” belonging to frontal and parietal areas of the brain. Their results are in general agreement with our results (Figures 6 & 7; Tables 2 & 3) (Zalesky et al., 2014). Specifically, MI and VI identified dynamic connections belonging to frontal and parietal areas (Figures 6 & 7 and Table 2), while the same metrics also identified parietal regions as having the highest dFC strength (Table 4), which is in agreement to Zalesky et al. (2014).

### 4.5 Surrogate data methodologies

A significant point in question was which methodology should be employed in order to produce randomized copies for hypothesis testing (surrogate data). There are two main approaches in the literaturewhich includes: Auto‐Regressive (AR) and Phase Randomization (PR) methods. Also, the AR method includes both a bivariate (Chang & Glover, 2010; Zalesky et al., 2014) and a multivariate (Liégeois et al., 2017) variant. The bivariate AR approach considers all pairs of time‐series and generates null data for each region combination separately. The bivariate AR procedure was examined in Zalesky et al. (2014), whereby it was reported that the null hypothesis of stationarity was successfully rejected for only ~4% of the examined pairs (293 out of 6,670). This result was also validated in a recent study utilizing bivariate AR, where it was reported that ~4.6% of the considered region pairs (306 out of 6,441) were detected as exhibiting dFC (Liégeois et al., 2017). However, the same study advised caution in the interpretation of results as derived from bivariate AR surrogates, as the latter may introduce false positives compared to MVAR surrogates. This was also shown using simulated data (Liégeois et al., 2017). Using real data along with the MVAR method, Liégeois et al. (2017) reported that less than 40 region pairs (out of 6,441) were identified as dynamically connected (Liégeois et al., 2017). In the current study, the MVAR method was implemented based on a 13‐variable MVAR model.

**Figure 8:**
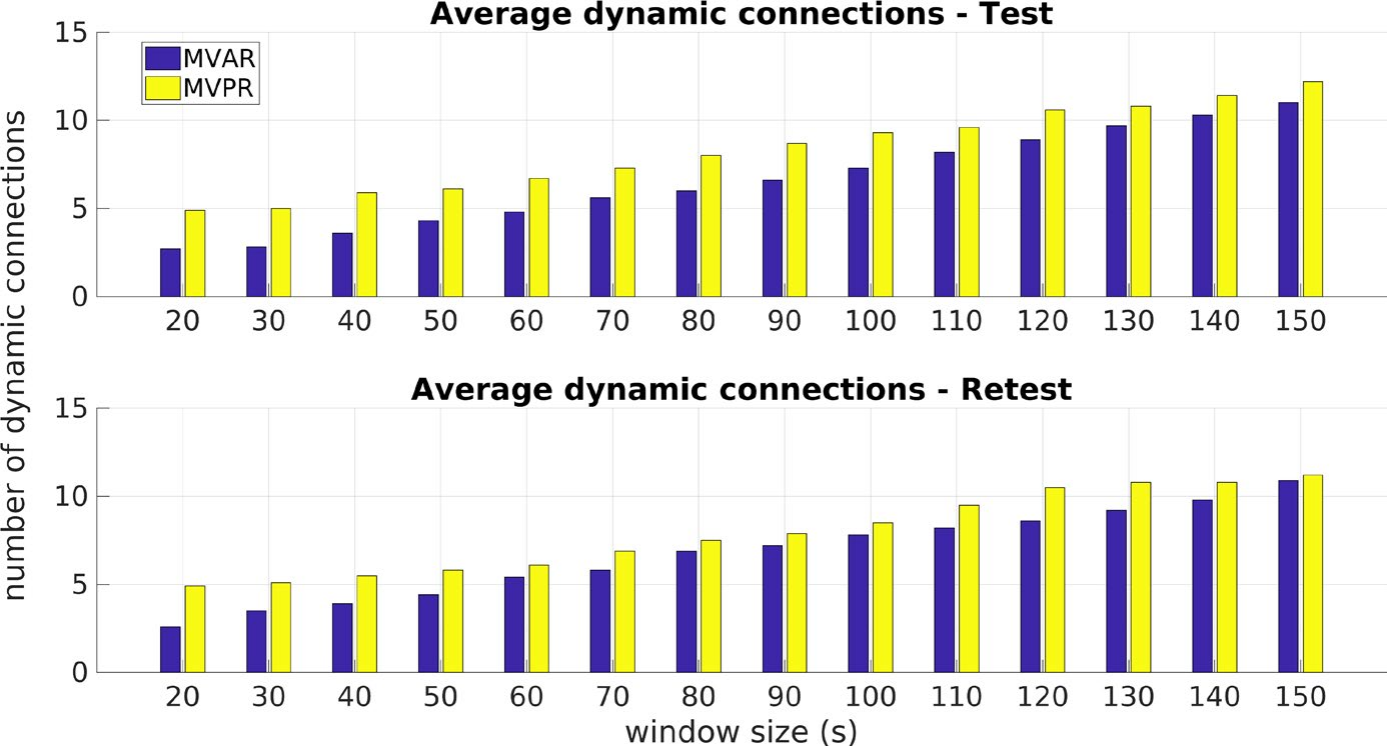
Average number of dynamically connected regions across all functional connectivity metrics, for each window size using the multivariate auto‐regressive and multivariate phase randomization surrogate methods

The rs‐fMRI literature has also employed the PR randomization framework for constructing null hypothesis data (Hindriks et al., 2016). This procedure considers a set of time‐series which are suitably processed in order to yield a set of randomized data, the auto‐covariance structure of which is the same as the initial data (Prichard & Theiler, 1994). As before, a bivariate or MVPR approach can be used for generating surrogate data. We primarily focused on the MVPR approach as it better preserved properties (auto‐covariance, stationary cross‐correlation, power spectral density, cross power spectral density, and amplitude distribution) of the initial data (Figure S2 in the Supporting information). Overall, the number of dynamically connected regions was found to be larger compared to the MVAR approach, which contradicts Liégeois et al. (2017), where it was found that the MVAR approach yielded more dynamic connections compared to the MVPR. This difference is mainly attributed to the MTD metric, as some discrepancies were observed compared to the MVAR approach. Figure 8 shows the average number of dynamic connections across all metrics for each examined window size. It can be seen that the MVPR method yields a larger average number of dynamically connected regions compared to MVAR.

### 4.6 Null hypothesis distributions

To conclude whether an examined region pair exhibits dFC, the definition of a null distribution through randomization of initial BOLD time‐series is required. In the present study, two options were implemented for hypothesis testing; one null distribution for each region pair and a single null distribution for all pairs. The latter was achieved by aggregating the 78 distributions into a single one consisting of 19,500 values. In Section S5 of Supporting information, examples of the resulting null hypotheses are provided for both cases, which generally yielded different results. Specifically, the usage of a single distribution for each region pair resulted in rejecting $H_{0}$ for almost all region pairs for small window sizes and for all region pairs using larger window sizes. This result was observed for all employed metrics. Therefore, the results presented above using a single null distributions support earlier studies for aggregating the individual null hypothesis distributions (Zalesky et al., 2014).

### 4.7 Study limitations

An important parameter of the sliding window methodology is the window size and previous studies reported how it affected the resulting windowed correlations relative to the frequency component of the initial signals (Leonardi & Van De Ville, 2015; Shakil et al., Shakil et al., 2015, 2018). In these latter studies, BOLD signals were modeled as a sum of sinusoidal signals and analytical derivation of their correlation was possible, resulting in a precise definition of the frequencies in each windowed correlation time‐series. Subsequent analysis suggested that the window size should be larger than the inverse frequency of the minimum frequency present in initial signals (Shakil et al., 2018, 2015). In the present study, such an approach was not considered for avoiding additional assumptions. Instead, we assessed the performance of different FC metrics with respect to the test–retest reproducibility of dFC estimates, as well as the resulting dynamically connected region pairs as compared to previous results (Chang & Glover, 2010; Zalesky et al., 2014).

The fact that Pearson linear and partial correlation metrics were found to be the least sensitive, in terms of identifying previously reported dynamic connections (Table 2) is remarkable since the majority of previous studies have relied on this metric to examine dFC. One possible reason for this could be that Pearson correlation quantifies linear correlations between the examined variables, a condition which may not be always met when examining BOLD signals, i.e., some regions of the brain may be nonlinearly correlated. The results of the present study suggest that Pearson correlation may yield decreased sensitivity when detecting dFC; however, they should not be interpreted as a negative criticism on studies using these metrics or (Leonardi & Van De Ville, 2015; Shakil et al., 2018, 2015), where a mathematical approach was employed, setting the basis for a more systematic and analytical approach for dFC estimation. We suggest that MI and VI should be used along with Pearson correlation and the obtained results should be reported and compared.

Moreover, MI and VI also have their own limitations. For instance, to obtain a reliable estimate, each window must contain an adequate number of data points. From the presented results (Table 2), it is suggested that at least 160≈120/TR (TR=0.72s) data points were able to yield a reliable estimate. In an experimental setting with a 5‐minute scanning session and a TR=2.5s, the total number of available data points would be 120. In this case, the usage of MI in sliding windows may not produce reliable estimates, due to the limited number of time points in each window, while the Pearson correlation may be more robust in the presence of a relatively low number of data points.

## 5 CONCLUSIONS

In this study, a thorough examination of dFC in the DMN using high quality rs‐fMRI data with sub‐second sampling rate was performed, by employing a sliding window approach along with 10 FC metrics over a wide range of window sizes. The purpose was to examine how different window sizes and FC metrics affect dFC assessment. To achieve this, a hypothesis testing framework was applied using surrogate data based on MVPR and MVAR approaches, focusing on the MVPR technique as it better preserved properties of the initial data. The obtained results suggest that MI and VI were able to yield more reproducible results in a test–retest analysis, as well as identify previously reported dynamic connections within the DMN, compared to alternative metrics, using a window size larger than 120 s. Additionally, since these two metrics have not been utilized in previous rs‐fMRI studies, as opposed to Pearson linear correlation, it is also suggested to jointly apply them and report their results.

## CONFLICT OF INTEREST

None declared.
