# Supplementary figures and images for: Assessment of dynamic functional connectivity in resting‐state fMRI using the sliding window technique
Source: Brain Behav. 2019 Mar 18;9(4):e01255. doi: 10.1002/brb3.1255 (PMC6456784; doi:10.1002/brb3.1255)

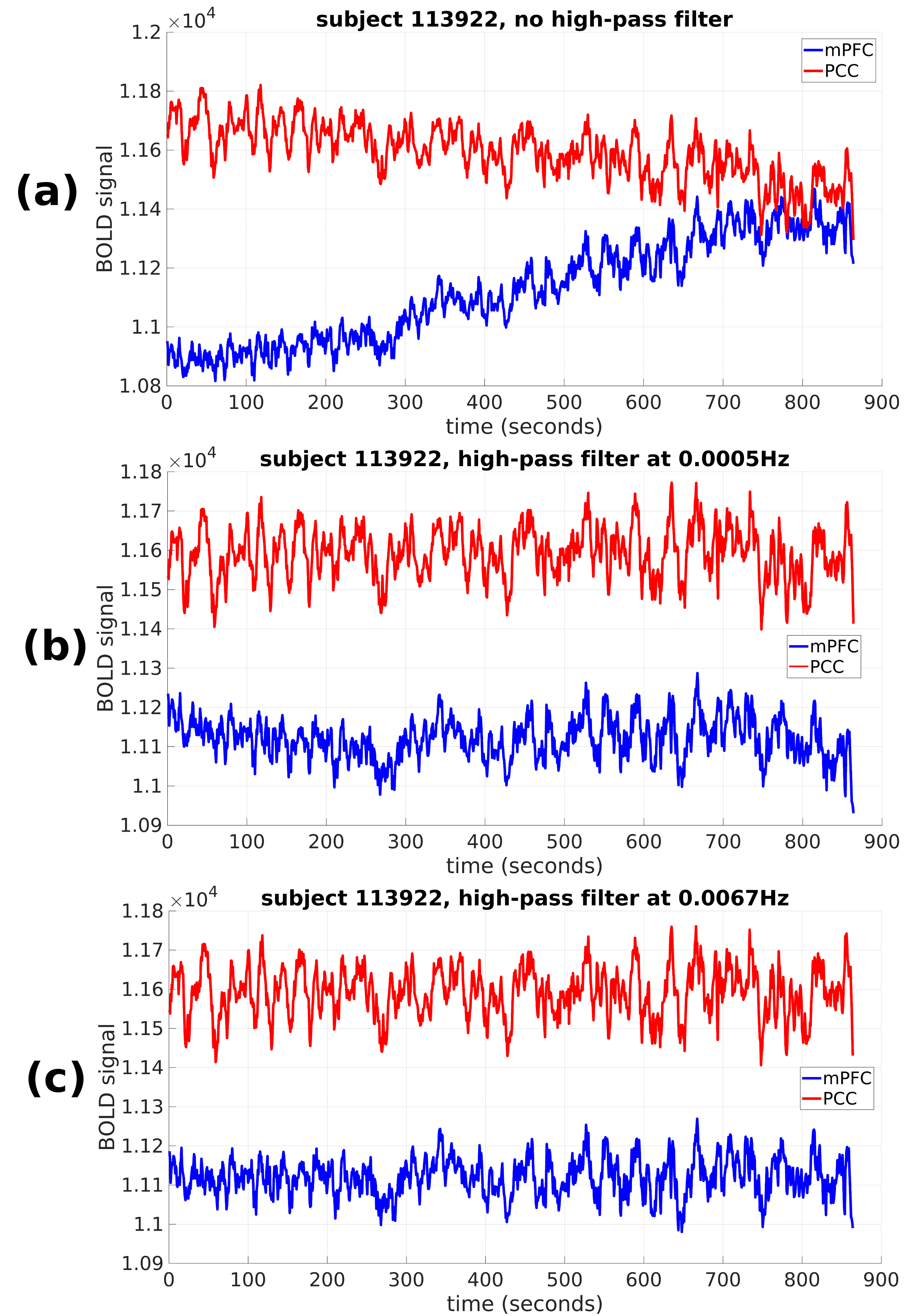

Supplement: Supplementary file 1 [file BRB3-9-e01255-s001.tif]

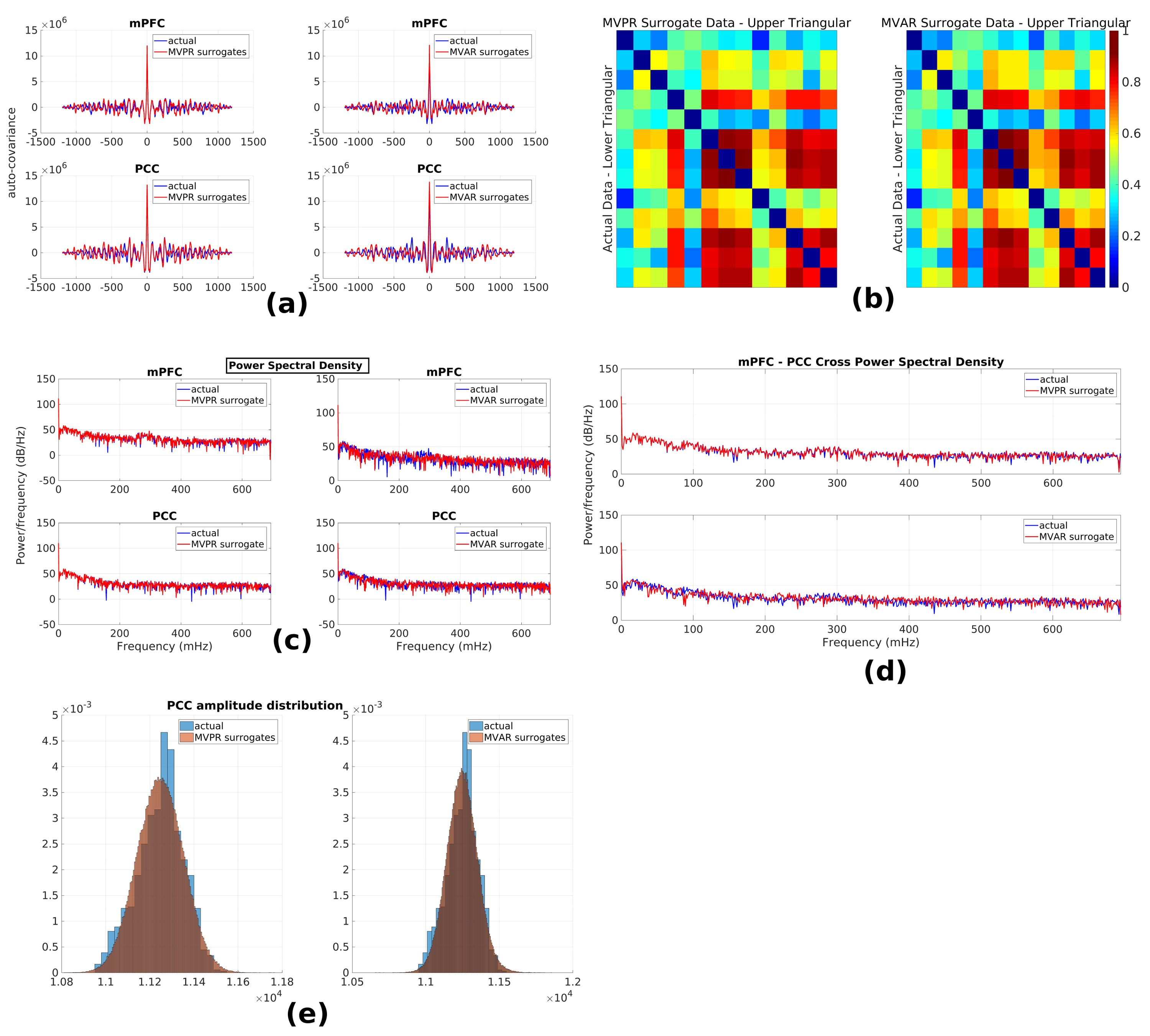

Supplement: Supplementary file 2 [file BRB3-9-e01255-s002.tif]

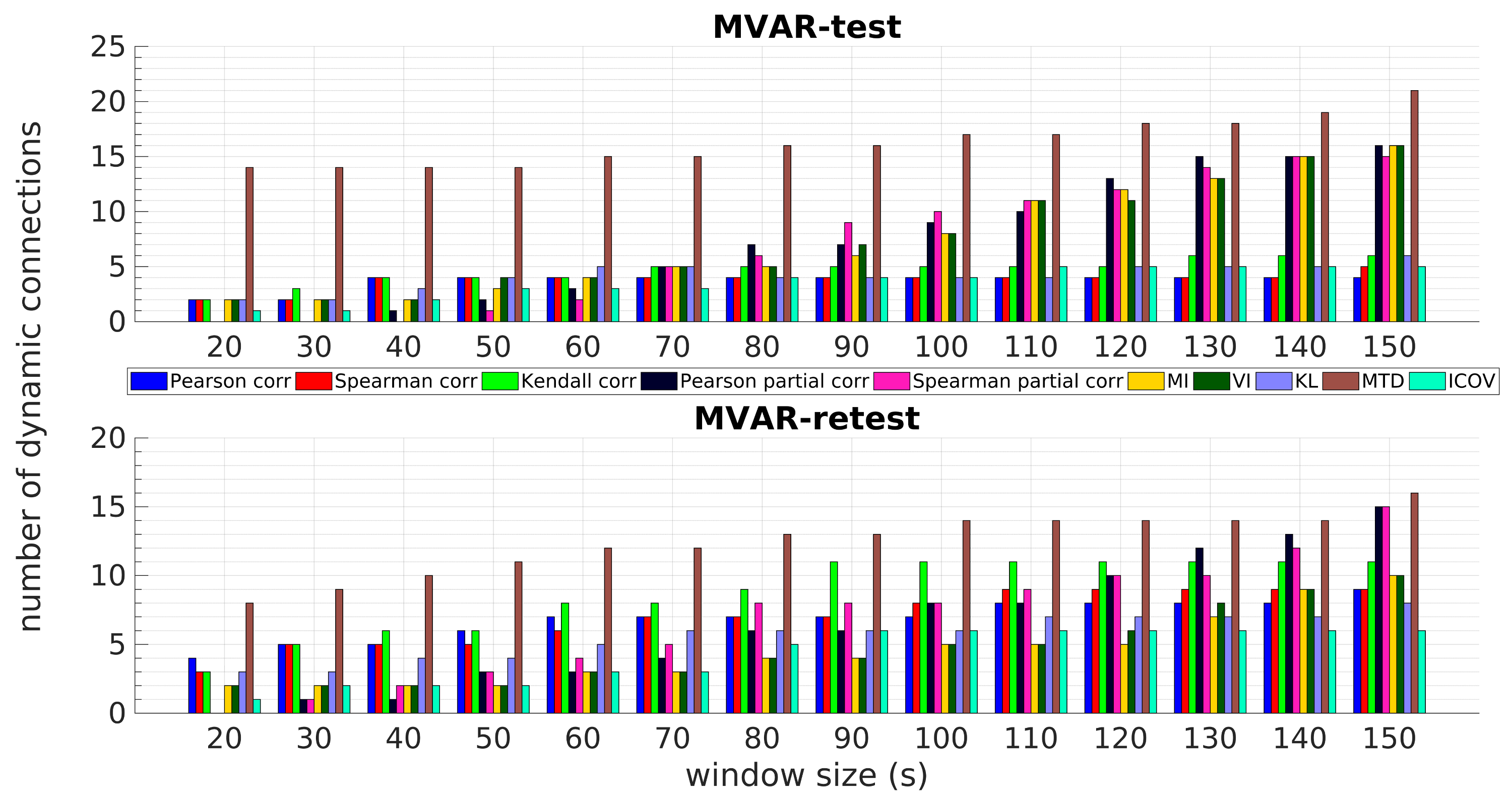

Supplement: Supplementary file 3 [file BRB3-9-e01255-s003.tif]

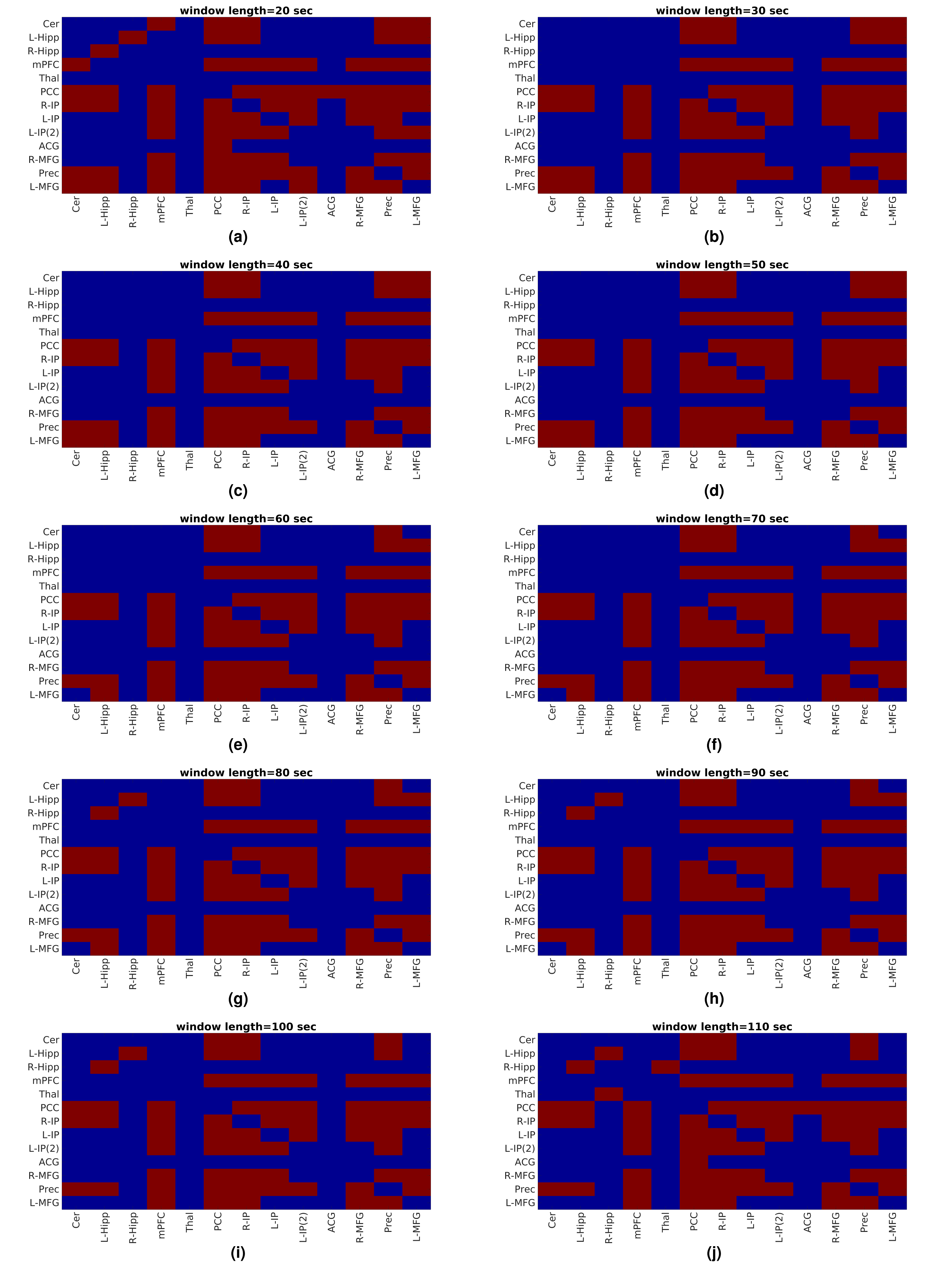

Supplement: Supplementary file 4 [file BRB3-9-e01255-s004.tif]

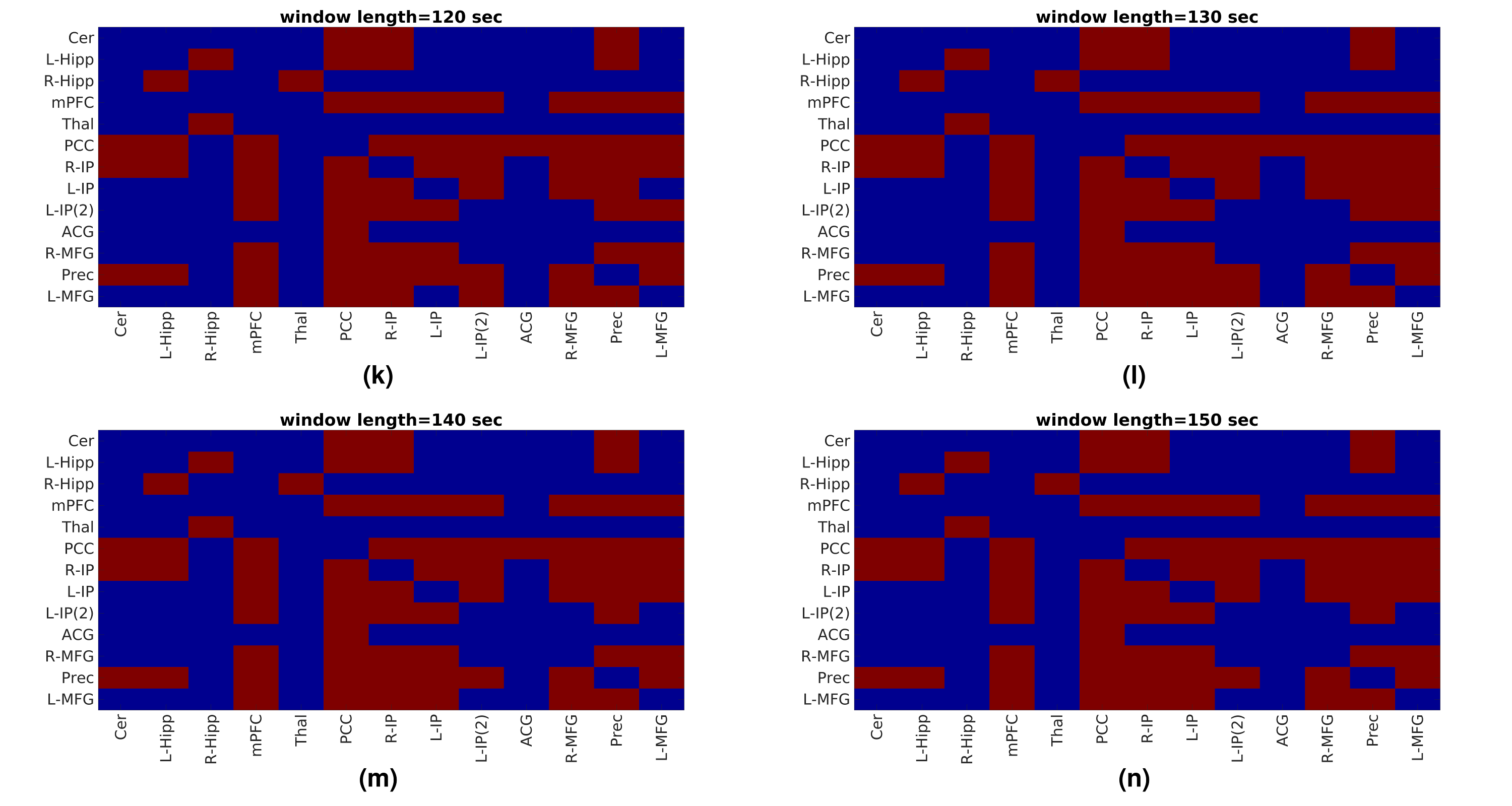

Supplement: Supplementary file 5 [file BRB3-9-e01255-s005.tif]

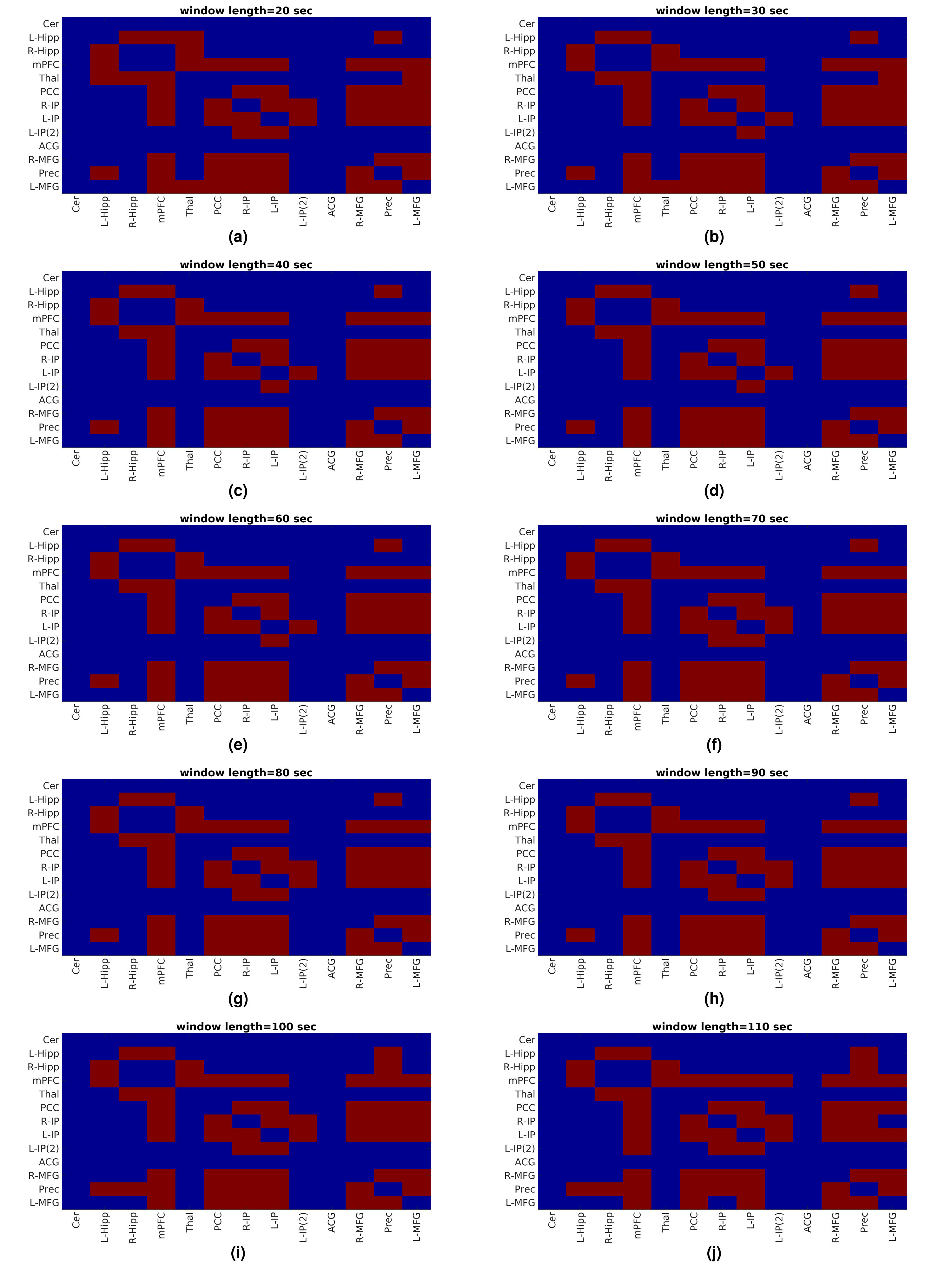

Supplement: Supplementary file 6 [file BRB3-9-e01255-s006.tif]

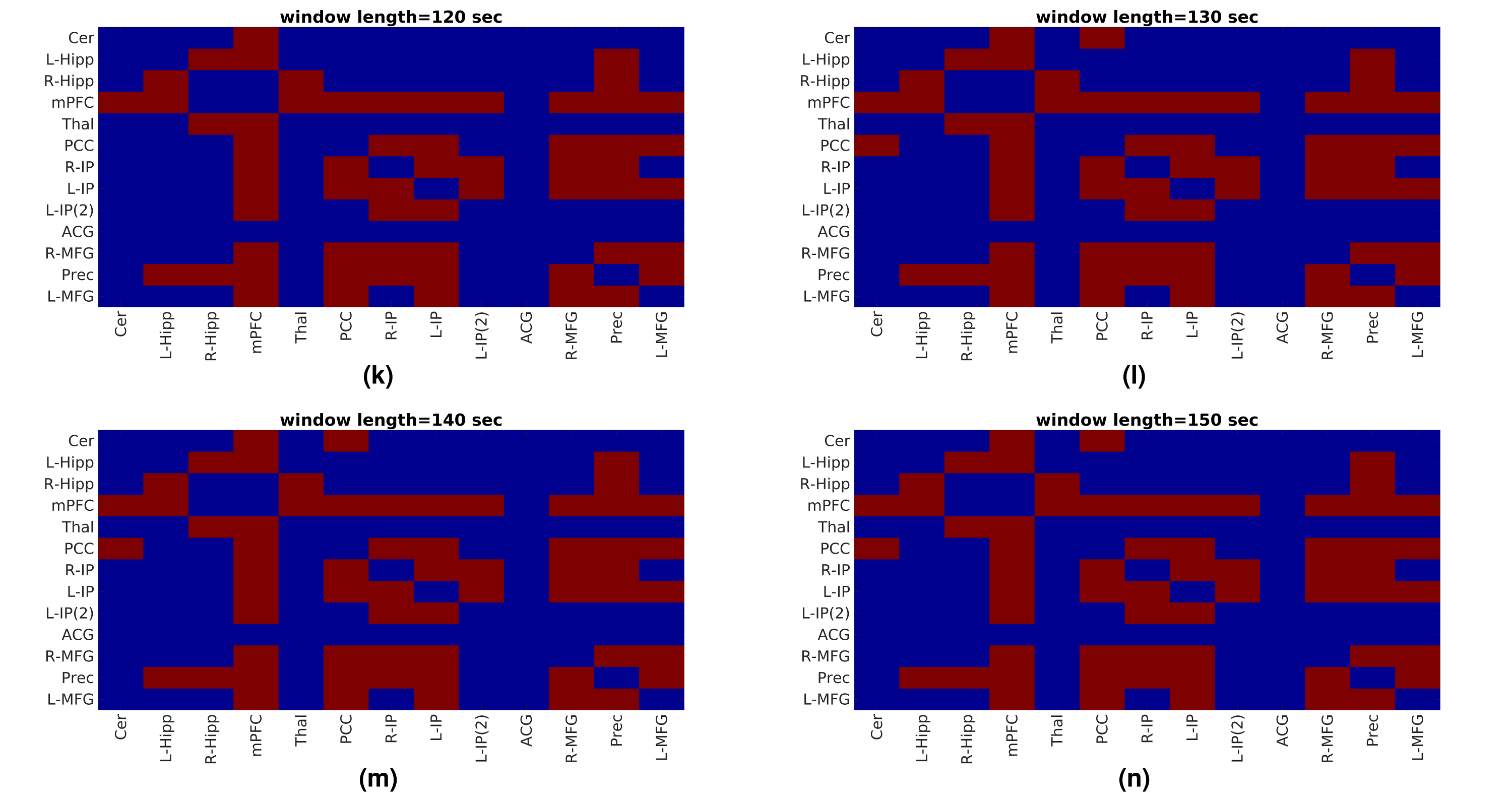

Supplement: Supplementary file 7 [file BRB3-9-e01255-s007.tif]

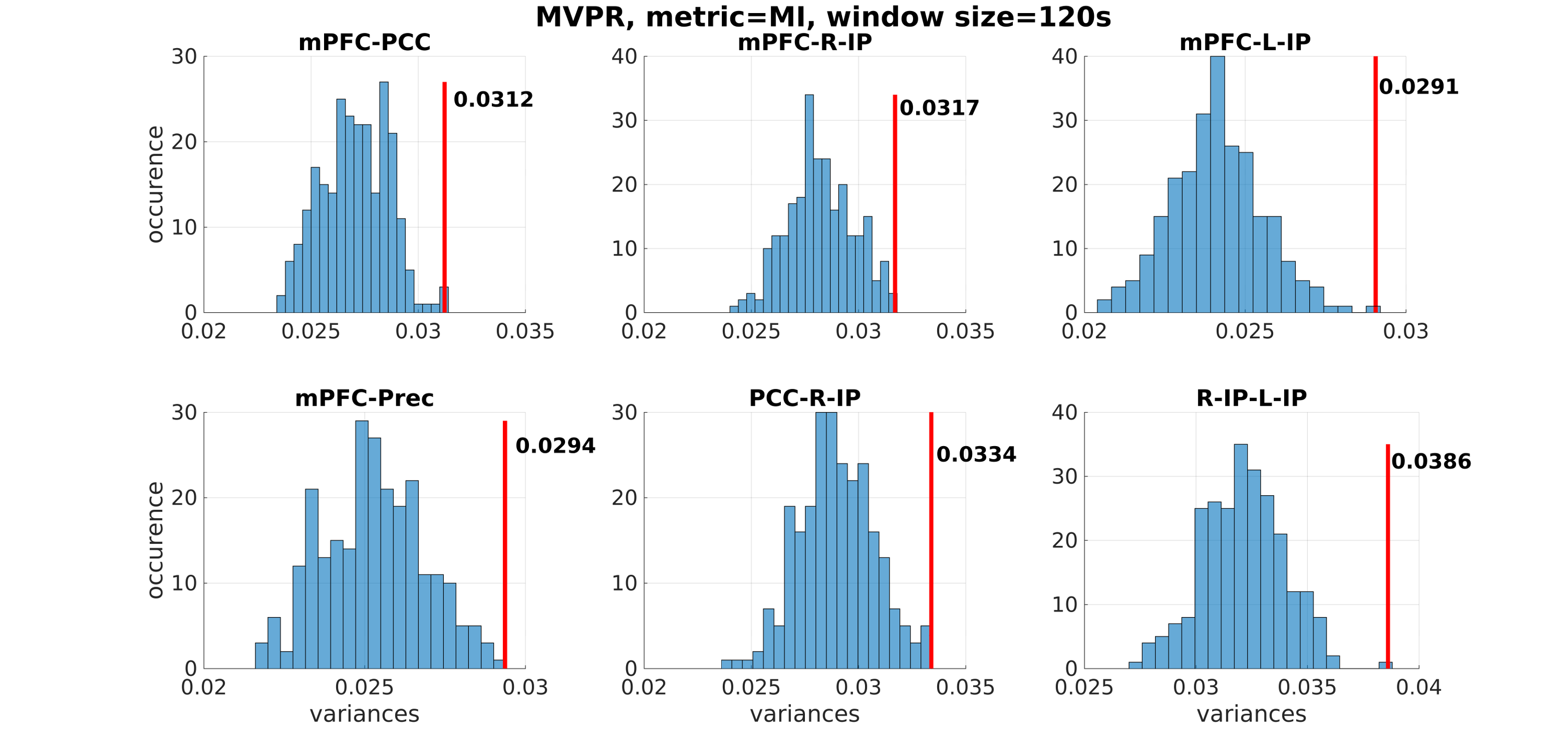

Supplement: Supplementary file 8 [file BRB3-9-e01255-s008.tif]

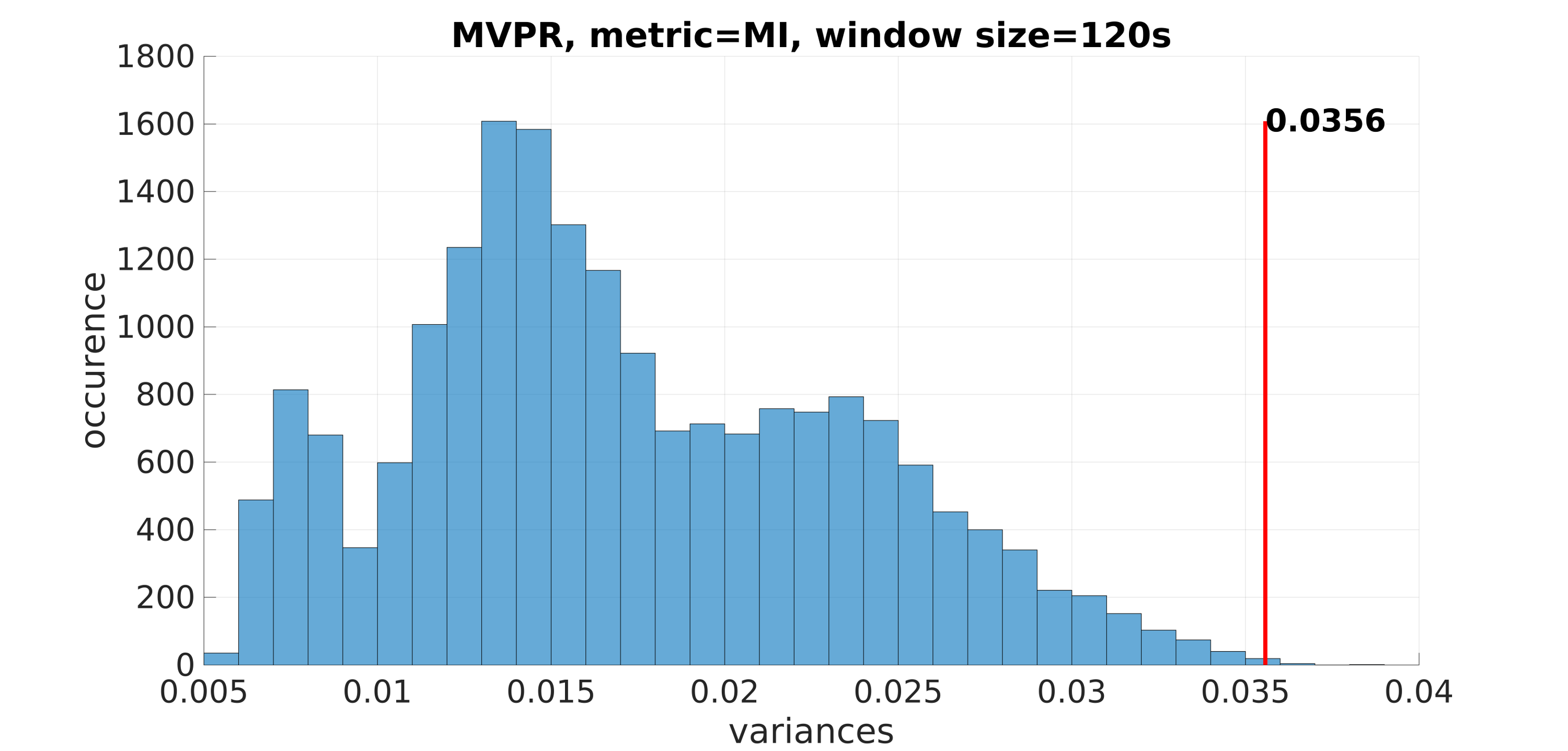

Supplement: Supplementary file 9 [file BRB3-9-e01255-s009.tif]
