# Supplementary material for: Assessment of dynamic functional connectivity in resting‐state fMRI using the sliding window technique
Source: Brain Behav. 2019 Mar 18;9(4):e01255. doi: 10.1002/brb3.1255 (PMC6456784; doi:10.1002/brb3.1255)
Supplement: Supplementary file 10 [file BRB3-9-e01255-s010.docx]

**Supporting Information**

## **High Pass Temporal Filtering**

Figure S1 shows an illustrative example (HCP 113922) of how the cutoff frequency in temporal filtering was chosen. In particular, Figure S1a displays the unfiltered time-series from mPFC and PCC, while Figure S1b-c illustrate the effects of temporal filtering using a cutoff frequency of $f=0.0005 Hz (2000s)$ and $f=0.0067 Hz (150s)$, respectively. Initially, the data were dominated by a linear trend which was removed using cutoff time of 2000s, in agreement to Stephen M. Smith et al (2013). However, higher order trends can be seen (i.e. mPFC – Figure S1b) which were sufficiently removed using a cutoff frequency of $f=0.0067 Hz (150s)$ (Figure S1c).

## **Surrogate Data Properties**

Figure S2a-e displays the preserved properties (auto-covariance, stationary cross-correlation, power spectral density, cross power spectral density and amplitude distribution), from the initial to surrogate data, using a representative subject (HCP 106319), for MVPR and MVAR techniques, respectively. As can be seen both MVPR and MVAR approaches produced surrogate data with well-matched properties, although the MVPR method seems to be more consistent in preserving these properties.

## **Dynamically connected region pairs using the MVAR framework**

For the construction of MVAR surrogate data, the model order $p$ was selected according to the minimization of Schwarz Bayesian Criterion (SBC) in the range $[1, 10]$. In most cases the finally selected order was $p=1$. In particular, for the 80% of the subjects a model of order 1 was fitted and for the rest a multivariate model of order 2 minimized the SBC.

Figure S3 shows the number of dynamic connections using the MVAR approach for all FC metrics, window sizes and test – retest datasets. As can be seen, an increasing window size resulted in more dynamically connected region pairs compared to a smaller one. In Table S1, dynamic connections with the PCC are illustrated (PCC – mPFC, PCC – R-IP and PCC – L-IP), which have been shown to exhibit time-varying connectivity patterns (Chang & Glover, 2010). Apart from MI and VI, the Pearson and Spearman partial correlations also identified some of the previously labeled dynamic connections at different window sizes, i.e. 140s and 120s, respectively. Additionally, Table S2 presents a detailed list of the dynamically connected regions, using the MVAR approach, similar to Table 3 of the manuscript. Finally, Table S3 lists the strength of dFC in descending order using the largest window size reported in third and fifth column of Table S2 (similar to Table 4 of the manuscript).

## **Dynamically connected region pairs using MTD and MVPR framework**

Due to the fact that multiple dynamically connected pairs were identified using the MVPR approach and the MTD metric, as well as this number did not increased with increasing window size as the remaining metrics (Figure 5 of the manuscript), the results are explicitly visualized for all window sizes, in Figure S4 and Figure S5, for the test and retest datasets, respectively, in the form of $13\times13$ matrices. Red color denotes the pair of regions exhibiting dFC and blue color indicates region pairs which were not found to be dynamically connected ($H_{0}$ could not be rejected).

## **Null Hypothesis Distribution**

Figure S6a shows some examples of the null distributions for selected region pairs in the case were each region combination has its own null distribution. Moreover, the thick red line indicates the percentile corresponding to a significance level of 0.05 (Bonferroni corrected) of the data, while these distributions were derived by using the MI metric at a window size of 120s. Additionally, Figure S6b showcases the single aggregated null distribution for MI using a window size of 120s.

## **References**

Chang, C., & Glover, G. H. (2010). Time–frequency dynamics of resting-state brain connectivity measured with fMRI. NeuroImage, 50(1), 81-98. doi: 10.1016/j.neuroimage.2009.12.011

Smith, S. M., Beckmann, C. F., Andersson, J., Auerbach, E. J., Bijsterbosch, J., Douaud, G., . . . Glasser, M. F. (2013). Resting-state fMRI in the Human Connectome Project. *NeuroImage, 80*, 144-168. doi: 10.1016/j.neuroimage.2013.05.039

**Supplementary Tables**

*Table S1: Connections with the PCC identified as dynamic using the sliding window approach for all examined FC metrics, using the MVAR surrogate method.*

| **MVAR** | | | | | | |
| --- | --- | --- | --- | --- | --- | --- |
| **Metric** | **PCC – mPFC** | | **PCC – R-IP** | | **PCC – L-IP** | |
|  | Dataset A | Dataset B | Dataset A | Dataset B | Dataset A | Dataset B |
| Pearson linear correlation | – | – | – | – | – | – |
| Pearson partial linear correlation | ≥ 110s | ≥ 100s | ≥ 120s | ≥ 140s | – | – |
| Inverse covariance matrix | ≥ 20s | ≥ 20s | – | – | – | – |
| Spearman rank correlation | – | – | – | – | – | – |
| Spearman partial rank correlation | ≥ 90s | ≥ 80s | ≥ 90s | – | – | ≥ 120s |
| Kendall correlation | – | – | – | ≥ 90s | – | – |
| Mutual Information | ≥ 100 | ≥ 150s | ≥ 90s | – | ≥ 140s | – |
| Variation of Information | ≥ 90 | ≥ 150s | ≥ 90s | – | ≥ 140s | – |
| Kullback-Leibler | – | – | – | – | – | – |
| Multiplication of Temporal Derivatives | ≥ 20s | ≥ 50s | ≥ 20s | ≥ 80s | ≥ 100s | ≥ 140s |

*Table S2: Dynamically connected region pairs identified using the sliding window and MVAR approach for different FC metrics and window sizes. In all cases, dFC between regions listed in the second and fourth column were detected for all window sizes larger than the value reported in the third and fifth column, respectively, unless indicated with “†”. In the latter case the “window size” column reports the range of window length whereby the corresponding regions yielded dFC.*

| **Metric** | **Dataset A** | | **Dataset B** | |
| --- | --- | --- | --- | --- |
|  | **Dynamically Connected Regions** | **Window Size (s)** | **Dynamically Connected Regions** | **Window Size (s)** |
| Pearson Linear Correlation | mPFC – R-IP | ≥ 20 | mPFC – R-IP | ≥ 20 |
|  | mPFC – L-IP | ≥ 40 | mPFC – L-IP | ≥ 20 |
|  | mPFC – Prec | ≥ 20 | mPFC – Prec | ≥ 20 |
|  | L-IP(2) – Prec | ≥ 40 | L-IP(2) – Prec | ≥ 20 |
|  | – | – | PCC – Prec | ≥ 50 |
|  | – | – | R-IP – L-IP(2) | ≥ 60 |
|  | – | – | L-IP – R-MFG | ≥ 110 |
|  | – | – | mPFC – L-MFG | ≥ 30 |
|  | – | – | R-MFG – Prec | ≥ 150 |
| Pearson Linear Partial Correlation | mPFC – PCC | ≥ 110 | mPFC – PCC | ≥ 100 |
|  | mPFC – R-IP | ≥ 50 | mPFC – R-IP | ≥ 50 |
|  | mPFC – L-IP | ≥ 70 | mPFC – L-IP | ≥ 70 |
|  | mPFC – R-MFG | ≥ 120 | mPFC – R-MFG | ≥ 80 |
|  | mPFC – Prec | ≥ 40 | mPFC – Prec | ≥ 30 |
|  | mPFC – L-MFG | ≥ 70 | mPFC – L-MFG | ≥ 100 |
|  | PCC – R-IP | ≥ 120 | PCC – R-IP | ≥ 140 |
|  | PCC – Prec | ≥ 60 | PCC – Prec | ≥ 50 |
|  | PCC – L-MFG | ≥ 100 | PCC – L-MFG | ≥ 120 |
|  | R-IP – Prec | ≥ 150 | R-IP – Prec | ≥ 80 |
|  | R-IP – L-MFG | ≥ 80 | R-IP – L-MFG | ≥ 120 |
|  | L-IP – Prec | ≥ 100 | L-IP – Prec | ≥ 150 |
|  | Prec – L-MFG | ≥ 80 | Prec – L-MFG | ≥ 150 |
|  | mPFC – L-IP(2) | ≥ 120 | R-IP – R-MFG | ≥ 130 |
|  | PCC – R-MFG | ≥ 130 | R-MFG – Prec | ≥ 130 |
|  | L-IP(2) – L-MFG | ≥ 130 | – | – |
| Inverse Covariance | mPFC – PCC | ≥ 20 | mPFC – PCC | ≥ 20 |
|  | mPFC – R-MFG | ≥ 40 | mPFC – R-MFG | ≥ 30 |
|  | R-IP – Prec | ≥ 110 | R-IP – Prec | ≥ 60 |
|  | L-IP – Prec | ≥ 50 | L-IP – Prec | ≥ 80 |
|  | R-IP – L-IP | ≥ 80 | L-IP – L-MFG | ≥ 80 |
|  | – | – | R-IP – R-MFG | ≥ 90 |
| Spearman Rank Correlation | mPFC – R-IP | ≥ 20 | mPFC – R-IP | ≥ 20 |
|  | mPFC – L-IP | ≥ 40 | mPFC – L-IP | ≥ 20 |
|  | mPFC – Prec | ≥ 20 | mPFC – Prec | ≥ 20 |
|  | PCC – Prec | ≥ 150 | PCC – Prec | ≥ 60 |
|  | L-IP(2) – Prec | ≥ 40 | L-IP(2) – Prec | ≥ 30 |
|  | – | – | PCC – R-IP | ≥ 110 |
|  | – | – | R-IP – L-IP(2) | ≥ 70 |
|  | – | – | L-IP – R-MFG | ≥ 100 |
|  | – | – | mPFC – L-MFG | ≥ 30 |
| Spearman Rank Partial Correlation | mPFC – PCC | ≥ 90 | mPFC – PCC | ≥ 80 |
|  | mPFC – R-IP | ≥ 60 | mPFC – R-IP | ≥ 40 |
|  | mPFC – L-IP | ≥ 110 | mPFC – L-IP | ≥ 60 |
|  | mPFC – R-MFG | ≥ 140 | mPFC – R-MFG | ≥ 70 |
|  | mPFC – Prec | ≥ 50 | mPFC – Prec | ≥ 30 |
|  | mPFC – L-MFG | ≥ 70 | mPFC – L-MFG | ≥ 80 |
|  | PCC – R-MFG | ≥ 130 | PCC – R-MFG | ≥ 150 |
|  | PCC – Prec | ≥ 70 | PCC – Prec | ≥ 50 |
|  | PCC – L-MFG | ≥ 100 | PCC – L-MFG | ≥ 140 |
|  | R-IP – Prec | ≥ 120 | R-IP – Prec | ≥ 80 |
|  | R-IP – L-MFG | ≥ 80 | R-IP – L-MFG | ≥ 140 |
|  | L-IP – Prec | ≥ 90 | L-IP – Prec | ≥ 110 |
|  | PCC – R-IP | ≥ 90 | PCC – L-IP | ≥ 120 |
|  | L-IP(2) – L-MFG | ≥ 130 | R-IP – R-MFG | ≥ 150 |
|  | Prec – L-MFG | ≥ 70 | R-MFG – L-MFG | ≥ 150 |
| Kendall Correlation | mPFC – R-IP | ≥ 20 | mPFC – R-IP | ≥ 20 |
|  | mPFC – L-IP | ≥ 30 | mPFC – L-IP | ≥ 20 |
|  | mPFC – Prec | ≥ 20 | mPFC – Prec | ≥ 20 |
|  | PCC – Prec | ≥ 70 | PCC – Prec | ≥ 30 |
|  | L-IP – R-MFG | ≥ 130 | L-IP – R-MFG | ≥ 90 |
|  | L-IP(2) – Prec | ≥ 40 | L-IP(2) – Prec | ≥ 40 |
|  | – | – | PCC – L-MFG | ≥ 60 |
|  | – | – | R-IP – L-IP(2) | ≥ 80 |
|  | – | – | R-MFG – Prec | ≥ 90 |
|  | – | – | mPFC – L-MFG | ≥ 30 |
|  | – | – | PCC – R-IP | ≥ 60 |
| Mutual Information | mPFC – PCC | ≥ 100 | mPFC – PCC | ≥ 150 |
|  | mPFC – R-IP | ≥ 60 | mPFC – R-IP | ≥ 100 |
|  | mPFC – L-IP | ≥ 120 | mPFC – Prec | ≥ 80 |
|  | PCC – Prec | ≥ 100 | PCC – Prec | ≥ 130 |
|  | R-IP – L-IP | ≥ 20 | R-IP – L-IP | ≥ 20 |
|  | R-IP – R-MFG | ≥ 110 | R-IP – R-MFG | ≥ 140 |
|  | R-IP – Prec | ≥ 20 | R-IP – Prec | ≥ 20 |
|  | L-IP – Prec | ≥ 50 | L-IP – Prec | ≥ 60 |
|  | R-MFG – Prec | ≥ 110 | R-MFG – Prec | ≥ 130 |
|  | Prec – L-MFG | ≥ 110 | Prec – L-MFG | ≥ 140 |
|  | mPFC – R-MFG | ≥ 140 | – | – |
|  | mPFC – Prec | ≥ 70 | – | – |
|  | PCC – R-IP | ≥ 90 | – | – |
|  | PCC – L-IP | ≥ 140 | – | – |
|  | R-IP – L-MFG | ≥ 130 | – | – |
|  | L-Hipp – Prec | ≥ 150 | – | – |
| Variation of Information | mPFC – PCC | ≥ 90 | mPFC – PCC | ≥ 150 |
|  | mPFC – R-IP | ≥ 50 | mPFC – R-IP | ≥ 100 |
|  | mPFC – Prec | ≥ 70 | mPFC – Prec | ≥ 80 |
|  | PCC – Prec | ≥ 100 | PCC – Prec | ≥ 120 |
|  | R-IP – L-IP | ≥ 20 | R-IP – L-IP | ≥ 20 |
|  | R-IP – R-MFG | ≥ 110 | R-IP – R-MFG | ≥ 130 |
|  | R-IP – Prec | ≥ 20 | R-IP – Prec | ≥ 20 |
|  | L-IP – Prec | ≥ 50 | L-IP – Prec | ≥ 60 |
|  | R-MFG – Prec | ≥ 110 | R-MFG – Prec | ≥ 130 |
|  | Prec – L-MFG | ≥ 110 | Prec – L-MFG | ≥ 140 |
|  | mPFC – L-IP | ≥ 130 | – | – |
|  | mPFC – R-MFG | ≥ 140 | – | – |
|  | PCC – R-IP | ≥ 90 | – | – |
|  | PCC – L-IP | ≥ 140 | – | – |
|  | R-IP – L-MFG | ≥ 130 | – | – |
|  | L-Hipp – Prec | ≥ 150 | – | – |
| Kullback-Leibler Divergence | mPFC – R-IP | ≥ 20 | mPFC – R-IP | ≥ 20 |
|  | mPFC – L-IP | ≥ 60 | mPFC – L-IP | ≥ 40 |
|  | mPFC – Prec | ≥ 50 | mPFC – Prec | ≥ 20 |
|  | mPFC – L-MFG | ≥ 150 | mPFC – L-MFG | ≥ 60 |
|  | R-IP – L-IP(2) (†) | [40, 70] | R-IP – L-IP(2) | ≥ 70 |
|  | L-IP(2) – Prec | ≥ 20 | L-IP(2) – Prec | ≥ 20 |
|  | R-IP – ACG | ≥ 120 | PCC – Prec | ≥ 110 |
|  | – | – | PCC – L-MFG | ≥ 150 |
| Multiplication of Temporal Derivatives | mPFC – PCC | ≥ 20 | mPFC – PCC | ≥ 50 |
|  | mPFC – R-IP | ≥ 20 | mPFC – R-IP | ≥ 20 |
|  | mPFC – L-IP | ≥ 60 | mPFC – L-IP | ≥ 20 |
|  | mPFC – R-MFG | ≥ 20 | mPFC – R-MFG | ≥ 20 |
|  | mPFC – Prec | ≥ 20 | mPFC – Prec | ≥ 20 |
|  | mPFC – L-MFG | ≥ 20 | mPFC – L-MFG | ≥ 20 |
|  | PCC – R-IP | ≥ 20 | PCC – R-IP | ≥ 80 |
|  | PCC – L-IP | ≥ 140 | PCC – L-IP | ≥ 100 |
|  | PCC – R-MFG | ≥ 100 | PCC – R-MFG | ≥ 150 |
|  | PCC – Prec | ≥ 20 | PCC – Prec | ≥ 40 |
|  | PCC – L-MFG | ≥ 20 | PCC – L-MFG | ≥ 30 |
|  | R-IP – L-IP | ≥ 20 | R-IP – L-IP | ≥ 20 |
|  | R-IP – R-MFG | ≥ 20 | R-IP – R-MFG | ≥ 20 |
|  | R-IP – Prec | ≥ 20 | R-IP – Prec | ≥ 60 |
|  | R-MFG – Prec | ≥ 120 | R-MFG – Prec | ≥ 150 |
|  | R-MFG – L-MFG | ≥ 150 | R-MFG – L-MFG | ≥ 20 |
|  | R-IP – L-IP(2) | ≥ 20 | – | – |
|  | R-IP – L-MFG | ≥ 20 | – | – |
|  | L-IP – Prec | ≥ 150 | – | – |
|  | Prec – L-MFG | ≥ 20 | – | – |
|  | Cer – R-IP | ≥ 80 | – | – |

*Table S3: DFC strength of significant edges in descending order, using the MVAR approach.*

| **Metric – Window Size (s)** | **Dataset A** | | **Dataset B** | |
| --- | --- | --- | --- | --- |
|  | **Dynamically Connected Regions** | **dFC Strength** | **Dynamically Connected Regions** | **dFC Strength** |
| Pearson Linear Correlation – 150 | mPFC–Prec | 0.0265 | mPFC–Prec | 0.0312 |
|  | mPFC–L-IP | 0.0245 | mPFC–R-IP | 0.0304 |
|  | L-IP(2)–Prec | 0.0241 | mPFC–L-MFG | 0.0255 |
|  | mPFC–R-IP | 0.0234 | mPFC–L-IP | 0.0241 |
|  | – | – | L-IP(2)–Prec | 0.0239 |
|  | – | – | R-IP–L-IP(2) | 0.0228 |
|  | – | – | PCC–Prec | 0.0223 |
|  | – | – | L-IP–R-MFG | 0.0211 |
|  | – | – | R-MFG–Prec | 0.0202 |
| Pearson Linear Partial Correlation – 150 | mPFC–R-IP | 0.0159 | mPFC–R-IP | 0.0164 |
|  | mPFC–Prec | 0.0159 | mPFC–Prec | 0.0159 |
|  | PCC–Prec | 0.0147 | PCC–Prec | 0.0149 |
|  | mPFC–L-MFG | 0.0142 | mPFC–L-IP | 0.0148 |
|  | R-IP–L-MFG | 0.0134 | mPFC–R-MFG | 0.0146 |
|  | Prec–L-MFG | 0.0131 | mPFC–PCC | 0.0140 |
|  | mPFC–R-MFG | 0.0128 | mPFC–L-MFG | 0.0135 |
|  | L-IP–Prec | 0.0126 | R-IP–L-MFG | 0.0132 |
|  | mPFC–L-IP | 0.0124 | R-IP–Prec | 0.0131 |
|  | PCC–L-MFG | 0.0124 | R-IP–R-MFG | 0.0130 |
|  | PCC–R-IP | 0.0118 | PCC–L-MFG | 0.0129 |
|  | mPFC–L-IP(2) | 0.0117 | PCC–R-IP | 0.0126 |
|  | mPFC–PCC | 0.0116 | R-MFG–Prec | 0.0125 |
|  | PCC–R-MFG | 0.0114 | L-IP–Prec | 0.0123 |
|  | R-IP–Prec | 0.0114 | Prec–L-MFG | 0.0123 |
|  | L-IP(2)–L-MFG | 0.0114 | – | – |
| Inverse Covariance – 110 | L-IP–Prec | 0.0383 | mPFC–PCC | 0.0387 |
|  | mPFC–PCC | 0.0347 | mPFC–R-MFG | 0.0365 |
|  | mPFC–R-MFG | 0.0342 | R-IP–Prec | 0.0363 |
|  | R-IP–L-IP | 0.0307 | L-IP–Prec | 0.0327 |
|  | R-IP–Prec | 0.0298 | R-IP–R-MFG | 0.0325 |
|  | – | – | L-IP–L-MFG | 0.0312 |
| Spearman Rank Correlation – 150 | mPFC–Prec | 0.0259 | mPFC–R-IP | 0.0297 |
|  | mPFC–L-IP | 0.0244 | mPFC–Prec | 0.0297 |
|  | L-IP(2)–Prec | 0.0236 | mPFC–L-MFG | 0.0250 |
|  | mPFC–R-IP | 0.0223 | mPFC–L-IP | 0.0234 |
|  | PCC–Prec | 0.0199 | L-IP(2)–Prec | 0.0226 |
|  | – | – | R-IP–L-IP(2) | 0.0221 |
|  | – | – | PCC–Prec | 0.0219 |
|  | – | – | L-IP–R-MFG | 0.0211 |
|  | – | – | PCC–R-IP | 0.0205 |
| Spearman Rank Partial Correlation – 150 | mPFC–Prec | 0.0155 | mPFC–R-IP | 0.0155 |
|  | mPFC–R-IP | 0.0147 | mPFC–Prec | 0.0155 |
|  | mPFC–L-MFG | 0.0143 | mPFC–PCC | 0.0147 |
|  | PCC–Prec | 0.0141 | mPFC–L-IP | 0.0146 |
|  | R-IP–L-MFG | 0.0136 | PCC–Prec | 0.0146 |
|  | Prec–L-MFG | 0.0131 | mPFC–R-MFG | 0.0140 |
|  | L-IP–Prec | 0.0129 | mPFC–L-MFG | 0.0134 |
|  | PCC–L-MFG | 0.0128 | PCC–L-IP | 0.0126 |
|  | mPFC–PCC | 0.0122 | R-IP–Prec | 0.0126 |
|  | PCC–R-IP | 0.0122 | L-IP–Prec | 0.0126 |
|  | mPFC–L-IP | 0.0120 | PCC–L-MFG | 0.0122 |
|  | mPFC–R-MFG | 0.0119 | R-IP–R-MFG | 0.0122 |
|  | R-IP–Prec | 0.0119 | R-IP–L-MFG | 0.0122 |
|  | PCC–R-MFG | 0.0117 | R-MFG–L-MFG | 0.0121 |
|  | L-IP(2)–L-MFG | 0.0116 | PCC–R-MFG | 0.0120 |
| Kendall Correlation – 130 | mPFC–Prec | 0.0162 | mPFC–Prec | 0.0172 |
|  | mPFC–L-IP | 0.0146 | mPFC–R-IP | 0.0167 |
|  | mPFC–R-IP | 0.0137 | mPFC–L-MFG | 0.0147 |
|  | PCC–Prec | 0.0133 | PCC–Prec | 0.0141 |
|  | L-IP(2)–Prec | 0.0132 | mPFC–L-IP | 0.0134 |
|  | L-IP–R-MFG | 0.0118 | PCC–R-IP | 0.0128 |
|  | – | – | R-IP–L-IP(2) | 0.0122 |
|  | – | – | L-IP–R-MFG | 0.0121 |
|  | – | – | L-IP(2)–Prec | 0.0121 |
|  | – | – | R-MFG–Prec | 0.0119 |
|  | – | – | PCC–L-MFG | 0.0116 |
| Mutual Information – 150 | R-IP–Prec | 0.0582 | R-IP–Prec | 0.0523 |
|  | L-IP–Prec | 0.0550 | R-IP–L-IP | 0.0478 |
|  | R-IP–L-IP | 0.0501 | L-IP–Prec | 0.0478 |
|  | mPFC–Prec | 0.0490 | mPFC–Prec | 0.0454 |
|  | PCC–Prec | 0.0471 | mPFC–R-IP | 0.0401 |
|  | R-MFG–Prec | 0.0458 | R-MFG–Prec | 0.0394 |
|  | Prec–L-MFG | 0.0452 | PCC–Prec | 0.0388 |
|  | PCC–R-IP | 0.0437 | Prec–L-MFG | 0.0386 |
|  | mPFC–R-IP | 0.0430 | R-IP–R-MFG | 0.0376 |
|  | R-IP–R-MFG | 0.0423 | mPFC–PCC | 0.0372 |
|  | mPFC–PCC | 0.0415 | – | – |
|  | mPFC–L-IP | 0.0401 | – | – |
|  | R-IP–L-MFG | 0.0399 | – | – |
|  | PCC–L-IP | 0.0396 | – | – |
|  | mPFC–R-MFG | 0.0393 | – | – |
|  | L-Hipp–Prec | 0.039 | – | – |
| Variation of Information – 150 | R-IP–Prec | 0.0568 | R-IP–Prec | 0.0507 |
|  | L-IP–Prec | 0.0542 | L-IP–Prec | 0.0469 |
|  | mPFC–Prec | 0.0486 | R-IP–L-IP | 0.0465 |
|  | R-IP–L-IP | 0.0486 | mPFC–Prec | 0.0448 |
|  | PCC–Prec | 0.0460 | mPFC–R-IP | 0.0393 |
|  | R-MFG–Prec | 0.0452 | R-MFG–Prec | 0.0390 |
|  | Prec–L-MFG | 0.0444 | PCC–Prec | 0.0385 |
|  | PCC–R-IP | 0.0430 | Prec–L-MFG | 0.0379 |
|  | mPFC–R-IP | 0.0424 | R-IP–R-MFG | 0.0373 |
|  | mPFC–PCC | 0.0411 | mPFC–PCC | 0.0366 |
|  | R-IP–R-MFG | 0.0411 | – | – |
|  | mPFC–L-IP | 0.0394 | – | – |
|  | R-IP–L-MFG | 0.0394 | – | – |
|  | PCC–L-IP | 0.0393 | – | – |
|  | L-Hipp–Prec | 0.0386 | – | – |
|  | mPFC–R-MFG | 0.0386 | – | – |
| Kullback-Leibler Divergence – 150 | mPFC–R-IP | 0.0115 | mPFC–R-IP | 0.0134 |
|  | mPFC–L-IP | 0.0105 | mPFC–Prec | 0.0120 |
|  | mPFC–Prec | 0.0100 | mPFC–L-IP | 0.0110 |
|  | L-IP(2)–Prec | 0.0097 | mPFC–L-MFG | 0.0108 |
|  | R-IP–ACG | 0.0091 | L-IP(2)–Prec | 0.0108 |
|  | mPFC–L-MFG | 0.0089 | R-IP–L-IP(2) | 0.0104 |
|  | – | – | PCC–L-MFG | 0.0096 |
|  | – | – | PCC–Prec | 0.0095 |
| Multiplication of Temporal Derivatives – 150 | PCC–R-IP | 0.0329 | mPFC–R-MFG | 0.0434 |
|  | R-IP–Prec | 0.0290 | mPFC–L-MFG | 0.0313 |
|  | mPFC–R-MFG | 0.0286 | PCC–Prec | 0.0206 |
|  | PCC–Prec | 0.0264 | R-IP–L-IP | 0.0201 |
|  | mPFC–R-IP | 0.0252 | mPFC–Prec | 0.0196 |
|  | mPFC–Prec | 0.0212 | mPFC–PCC | 0.0192 |
|  | mPFC–PCC | 0.0205 | R-MFG–L-MFG | 0.0188 |
|  | mPFC–L-MFG | 0.0183 | mPFC–R-IP | 0.0177 |
|  | R-IP–L-MFG | 0.0179 | PCC–L-MFG | 0.0177 |
|  | R-IP–L-IP | 0.0177 | mPFC–L-IP | 0.0174 |
|  | R-IP–L-IP(2) | 0.0156 | R-IP–Prec | 0.0154 |
|  | R-IP–R-MFG | 0.0153 | R-IP–R-MFG | 0.0147 |
|  | mPFC–L-IP | 0.0152 | PCC–R-IP | 0.0146 |
|  | Prec–L-MFG | 0.0152 | PCC–L-IP | 0.0144 |
|  | PCC–L-MFG | 0.0150 | PCC–R-MFG | 0.0131 |
|  | PCC–R-MFG | 0.0136 | R-MFG–Prec | 0.0129 |
|  | R-MFG–Prec | 0.0135 | – | – |
|  | PCC–L-IP | 0.0127 | – | – |
|  | L-IP–Prec | 0.0126 | – | – |
|  | Cer–R-IP | 0.0124 | – | – |
|  | R-MFG–L-MFG | 0.0123 | – | – |

**Supplementary Figure Legends**

Figure S1: Effect of high-pass temporal filtering, using a representative subject (HCP 113922), with (a) no high-pass filtering, (b) cutoff frequency at 0.0005Hz (cutoff time 2000s) and (c) cutoff frequency at 0.0067Hz (cutoff time 150s). A very low cutoff frequency, e.g. 0.0005Hz, resulted in removing linear trends from the data, while a larger one, e.g. 0.0067Hz, sufficiently removed higher order trends.

**Figure S2:** Properties of initial data that were preserved in surrogate time-series using the MVPR and MVAR approaches: (a) auto-covariance, (b) stationary cross-correlation, (c) power spectral density, (d) cross power spectral density and (e) amplitude distribution, from a representative subject (HCP 106319). In (b), the top (left) rows (columns) represent regions with the following order: Cer: Cerebellum, L-Hipp: Left Hippocampus, R-Hipp: Right Hippocampus, mPFC: Medial Prefrontal Cortex, Thal: Thalamus, PCC: Posterior Cingulate Cortex, R-IP: Right Inferior Parietal, L-IP: Left Inferior Parietal, L-IP(2): Left Inferior Parietal – 2, ACG: Anterior Cingulate Gyrus, R-MFG: Right Middle Frontal Gyrus, Prec: Precuneus, L-MFG: Left Middle Frontal Gyrus.

Figure S3: Number of dynamically connected regions for all FC metrics and window sizes with the MVAR approach, using test (upper) and retest (lower) datasets. An increasing window size yielded in more dynamically connected ROIs for all metrics compared to a shorter one. *Metric abbreviations:* MI: mutual information, VI: variation of information, KL: Kullback-Leibler divergence, MTD: multiplication of temporal derivatives, ICOV: inverse covariance

**Figure S4:** Dynamically connected region pairs using the MVPR framework and MTD metric, for the test dataset for all window sizes, from (a) 20s to (n) 150s, with a step of 10s.

**Figure S5:** Dynamically connected region pairs using the MVPR framework and MTD metric, for the retest dataset for all window sizes, from (a) 20s to (n) 150s, with a step of 10s.

**Figure S6:** Null hypothesis distributions using the MVPR surrogate data method, for MI and window size of 120s, while employing (a) a single distribution for each pair and (b) a single null distribution for all examined pairs. The thick red vertical line indicates the corresponding significance level of 0.05 (Bonferroni corrected). Horizontal axis refers to variance values, while vertical axis displays the occurrences for each histogram bin. Distributions in (a) are plotted for selected region pairs. *Regions abbreviations:* mPFC: medial Prefrontal Cortex, PCC: Posterior Cingulate Cortex, R-IP: Right Inferior Parietal, L-IP: Left Inferior Parietal, Prec: Precuneus.
